# The chloroplast genome evolution of Venus slipper (*Paphiopedilum*): IR expansion, SSC contraction, and highly rearranged SSC regions

**DOI:** 10.1186/s12870-021-03053-y

**Published:** 2021-05-31

**Authors:** Yan-Yan Guo, Jia-Xing Yang, Ming-Zhu Bai, Guo-Qiang Zhang, Zhong-Jian Liu

**Affiliations:** 1 College of Plant Protection, Henan Agricultural University, Zhengzhou, 450002 China; 2 Key Laboratory of National Forestry and Grassland Administration for Orchid Conservation and Utilization, Shenzhen Key Laboratory for Orchid Conservation and Utilization, The National Orchid Conservation Center of China, The Orchid Conservation and Research Center of Shenzhen, Shenzhen, 518114 China; 3 Key Laboratory of National Forestry and Grassland Administration for Orchid Conservation and Utilization At College of Landscape Architecture, Fujian Agriculture and Forestry University, Fuzhou, 350002 China; 4 College of Forestry, Fujian Colleges and Universities Engineering Research Institute of Conservation and Utilization of Natural Bioresources, Fujian Agriculture and Forestry University, Fuzhou, 350002 China

**Keywords:** Orchidaceae, *Paphiopedilum*, Phylogenomics, Plastome, Boundary shift, IR, SSC boundary, Gene loss, Pseudogenization

## Abstract

**Background:**

*Paphiopedilum* is the largest genus of slipper orchids. Previous studies showed that the phylogenetic relationships of this genus are not well resolved, and sparse taxon sampling documented inverted repeat **(**IR) expansion and small single copy (SSC) contraction of the chloroplast genomes of *Paphiopedilum*.

**Results:**

Here, we sequenced, assembled, and annotated 77 plastomes of *Paphiopedilum* species (size range of 152,130 – 164,092 bp). The phylogeny based on the plastome resolved the relationships of the genus except for the phylogenetic position of two unstable species. We used phylogenetic and comparative genomic approaches to elucidate the plastome evolution of *Paphiopedilum*. The plastomes of *Paphiopedilum* have a conserved genome structure and gene content except in the SSC region. The large single copy/inverted repeat (LSC/IR) boundaries are relatively stable, while the boundaries of the inverted repeat and small single copy region (IR/SSC) varied among species. Corresponding to the IR/SSC boundary shifts, the chloroplast genomes of the genus experienced IR expansion and SSC contraction. The IR region incorporated one to six genes of the SSC region. Unexpectedly, great variation in the size, gene order, and gene content of the SSC regions was found, especially in the subg. *Parvisepalum*. Furthermore, *Paphiopedilum* provides evidence for the ongoing degradation of the *ndh* genes in the photoautotrophic plants. The estimated substitution rates of the protein coding genes show accelerated rates of evolution in *clpP*, *psbH*, and *psbZ*. Genes transferred to the IR region due to the boundary shift also have higher substitution rates.

**Conclusions:**

We found IR expansion and SSC contraction in the chloroplast genomes of *Paphiopedilum* with dense sampling, and the genus shows variation in the size, gene order, and gene content of the SSC region. This genus provides an ideal system to investigate the dynamics of plastome evolution.

**Supplementary Information:**

The online version contains supplementary material available at 10.1186/s12870-021-03053-y.

## Background

High throughput sequencing technology has made obtaining chloroplast genome (plastome) sequences more practical [1]. Many studies have used plastome data to address the phylogenetic relationships among land plants, chloroplast genome evolution, and patterns and rates of nucleotide substitutions [2–5]. These studies indicate that the chloroplast genome has striking variations in genome size, genome structure, and gene substitution rate across the angiosperms.

Though the average chloroplast genome of land plants is 151 kb, the average inverted repeat **(**IR) region is 25 kb [6, 7]. Published studies show extensive variation in the plastome size and length of the IR region [e.g. 4, 8]. The plastome size of *Pelargonium transvaalense* is 242,575 bp, with an IR region of 87,724 bp [8]. On the other hand, IR region loss has been detected in all lineages across land plants [9]. High throughout sequencing provides a good opportunity to test the plastome evolution in more groups.

*Paphiopedilum* (Venus slipper) is the largest genus of slipper orchids, mainly distributed in southeast Asia, with half of these species growing on islands. The sequenced plastomes of *Paphiopedilum* show the expansion of the IR region, while the SSC regions are greatly reduced in size and gene content [10–13]. Even typical SSC genes such as *ycf1*, *psaC*, and *ndhD* have been transferred to the IR region in *Paphiopedilum armeniacum* [10]. However, previous studies of *Paphiopedilum* plastomes are based on sparse taxon sampling, and the pattern of IR expansion/SSC contraction at the genus level are unknown. *Paphiopedilum* provides a unique opportunity to study the dynamics of the boundary shift impact on plastid genome structure and sequence evolution.

Additionally, there are still unresolved phylogenetic questions in *Paphiopedilum*. For instance, previous chloroplast markers cannot solve the deep phylogenetic relationship [14–16]. Resolving relationships in *Paphiopedilum* will promote the study of speciation of the genus. Recent phylogenetic analyses indicated widespread reticulate evolution of the genus and that sect. *Cochlopetalum* is not monophyletic in the chloroplast gene tree [14, 15]. Besides, there is also systematic uncertainty of four taxa (*P. canhii*, *P. fairrieanum*, *P. hirsutissimum*, and *P. rungiyasanum*) with unusual morphologies that fall outside the established section/subgenera groupings [15, 17]. For example, *P. canhii* is a newly described species from Vietnam, proposed to the subgeneric status [18, 19]. Given that *Paphiopedilum* is a genus with reticulate evolution and clonal propagation, current phylogenetic studies of the genus are rather limited and the effect of these events on the evolution of the chloroplast genome is unknown. *Paphiopedilum* is a suitable system to study the evolution of the chloroplast genome and to test whether whole plastome sequences can resolve the phylogeny of the genus.

In an effort to answer these unresolved questions, we used a genome skimming method to sequence 77 chloroplast genomes of *Paphiopedilum*. We used comparative genomics to study the evolution of the *Paphiopedilum* chloroplast genome and included four *Paphiopedilum* plastome sequences reported in previous studies [10, 12, 13, 20]. We analysed the sequences of all the shared protein coding genes from 81 *Paphiopedilum* samples to study patterns of evolutionary rates of the chloroplast genome. The goals of this study are to 1) reconstruct the phylogenetic relationships of the genus, especially the systematic positions of the phylogenetically unstable taxa (*P. canhii*, *P. fairrieanum*, *P. hirsutissimum*, and *P. rungiyasanum*), and test whether the chloroplast genome could resolve the recently diverged taxa of the genus; 2) characterize the chloroplast genome evolution pattern of *Paphiopedilum*; and 3) calculate the substitution rate of the coding genes, evaluate the impact of IR/SSC boundary shift, and test whether the genes transferred to the IR region due to boundary shift have decreased substitution rates.

## Results

### Plastomes of *Paphiopedilum*

We obtained 66 full plastome sequences. The other 11 plastome sequences had one or two gaps located in regions of high AT content within the three intergeneric regions (*trnS*-*trnG*, *trnE*-*trnT*, and *trnP*-*psaJ*). The obtained plastid genome sequences were deposited in GenBank (accession Nos. MN587749 – MN587825) (Table S1). The mean coverage depth of the sequenced plastomes was over 3000-fold (Table S1). We included four published plastome sequences, namely, *P. armeniacum* [10], *P. dianthum* [12], *P. malipoense* [20], and *P. niveum* [11], in subsequent analyses, yielding a total of 81 genomes of the genus *Paphiopedilum*. The downloaded plastome sequence of *Phragmipedium longifolium* [10] was used as the outgroup.

**Fig. 1:**
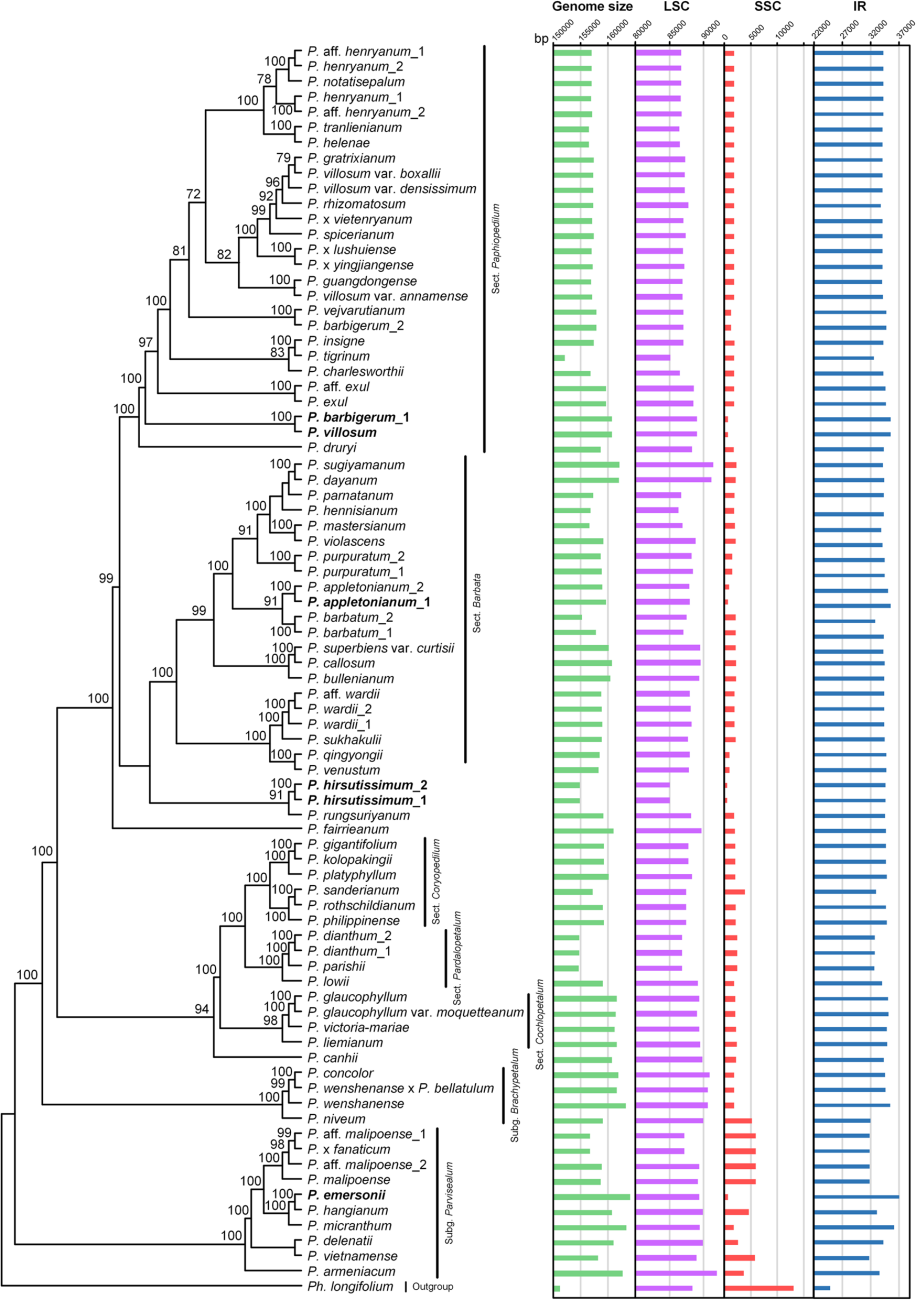
The length of plastid genome size, LSC, SSC, and IR regions were plotted on the ML tree of *Paphiopedilum*. LSC, SSC, and IR regions were scaled differently. The six samples in bold only have *trnL*-*UAG* preserved in the SSC region

**Fig. 2:**
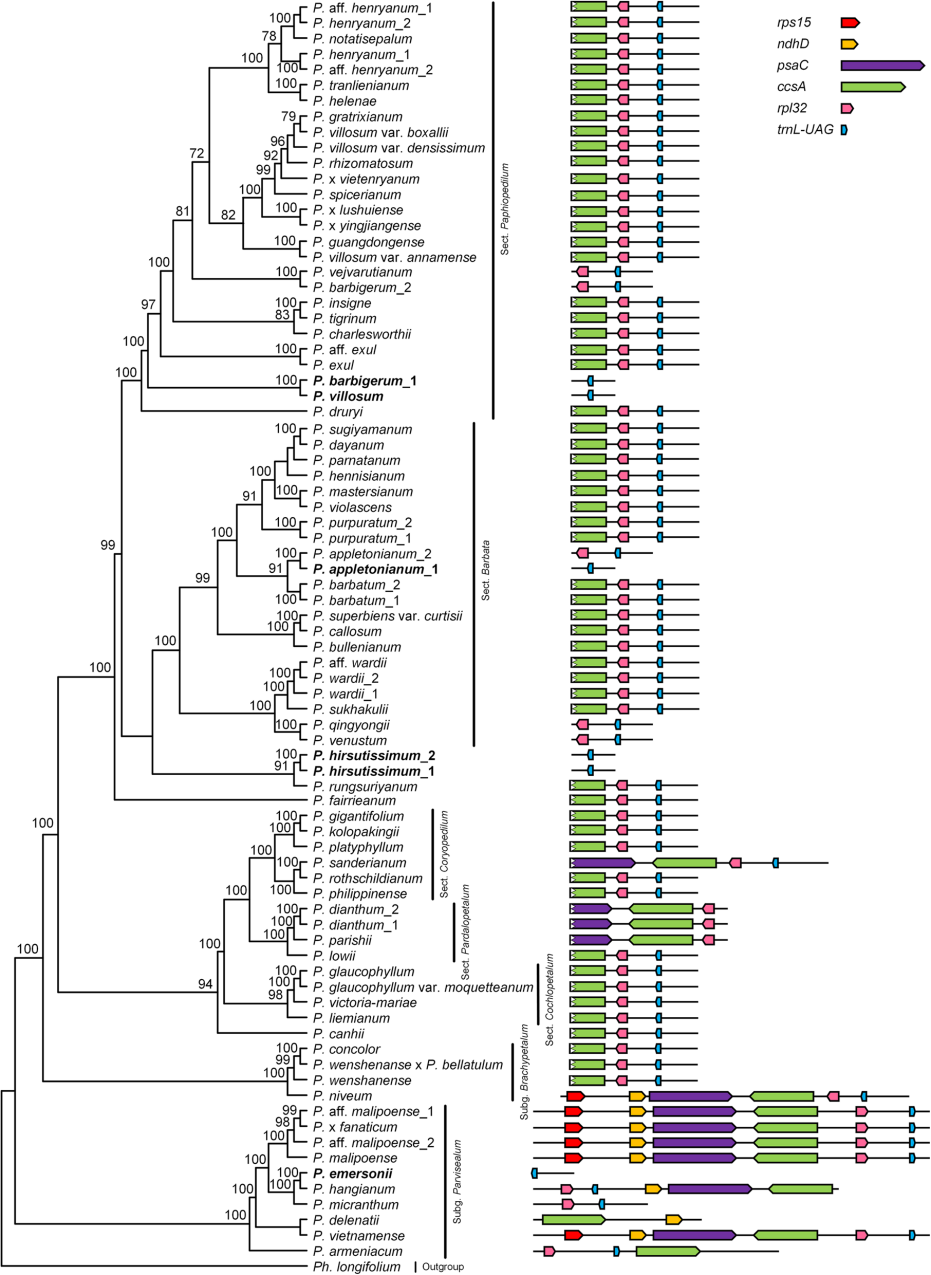
The variation in SSC regions of *Paphiopedilum*. The scaled representations of SSC types are plotted on the ML tree. The five species in bold lost all the functional *ndh* genes

**Fig. 3:**
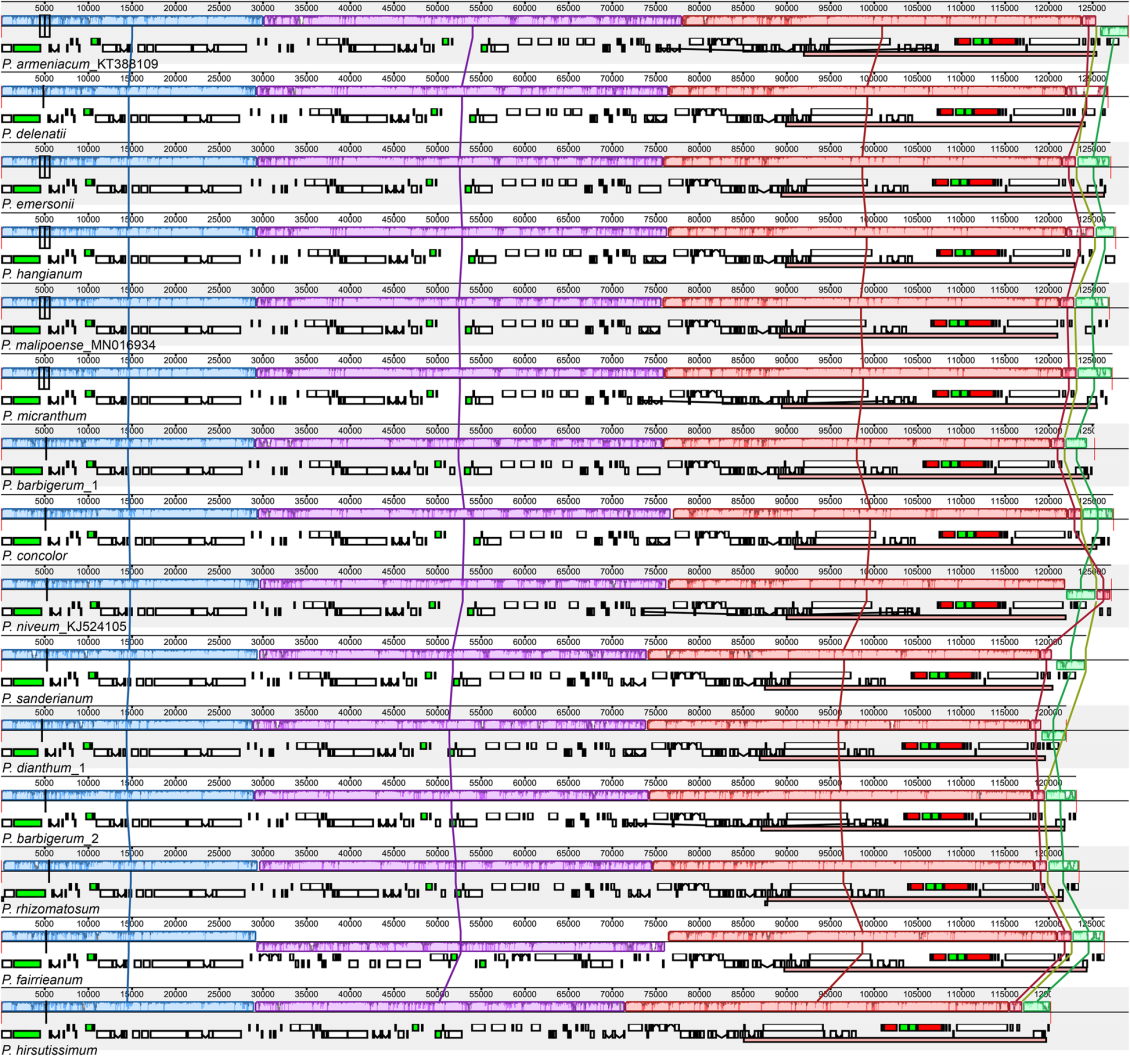
Extent of the gene rearrangements of 15 *Paphiopedilum* chloroplast genomes. Locally collinear blocks of the sequences are colour-coded and connected by lines

**Fig. 4:**
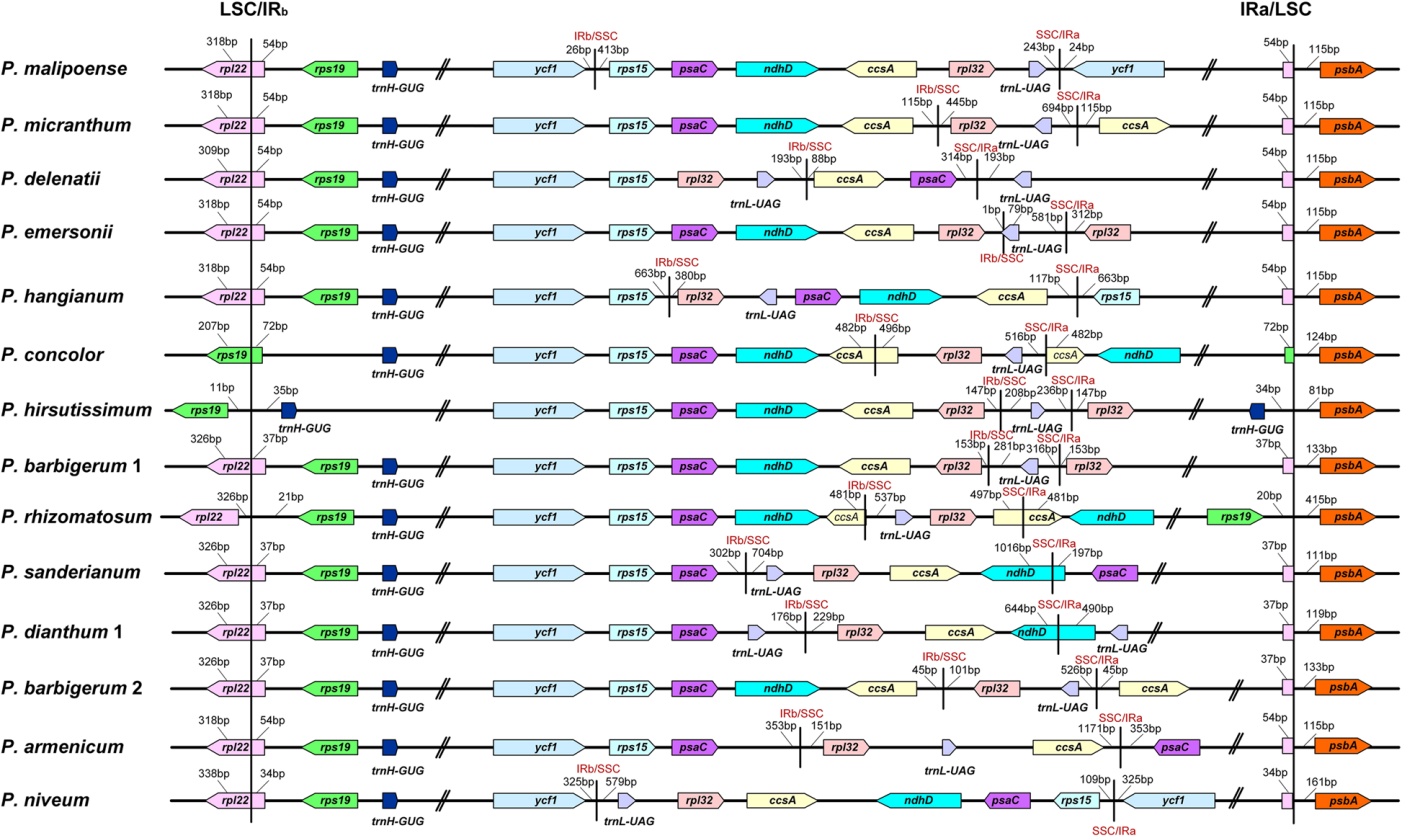
Shift of the LSC/IR boundaries and IR/SSC boundaries of *Paphiopedilum*

The genome size of *Paphiopedilum* ranged from 152,130 bp in *P. tigrinum* to 164,092 bp in *P. emersonii* (Table S1), *P. emersonii* had the largest number of genes (134 genes) and one of the shortest SSC regions (660 bp). The plastid genome of the genus shows typical quadripartite structure, with two identical copies of IR separated by a LSC region and an SSC region (Fig. S1). Compared with the plastome of other angiosperms, *Paphiopedilum* has numerous expansions of IRs. The length of the IR region was enlarged to 31,743 bp – 37,043 bp, with the length of the IR region of eight samples larger than 35 kb (Table S1), while the length of the SSC region contracted to 524 bp – 5916 bp (Fig. 1, Table S1). In addition, the SSC regions of *Paphiopedilum* are hotspots for gene transfer, loss, and rearrangement (Figs. 2, 3 and 4). The size variation in the SSC region mainly results from the transfer of typical SSC genes to IR regions and the loss/pseudogenization of *ndh* genes. Subg. *Parvisepalum* has a relatively larger SSC region than the other species in the genus (Figs. 1 and 2, Table S1). Pearson’s correlation analysis suggested that the length of IR region and SSC region are strongly correlated (*r* = – 0.8050, *P* < 0.001).

The gene order was conserved and composed of 127 to 134 genes, including 76 to 81 protein coding genes, 38 to 39 tRNA genes, eight rRNAs, three to eight pseudogenes, and 20 to 25 genes was duplicated in the IR region (Tables S1 and S2). In addition to the duplication of the IR regions, *trnG-GCC* was duplicated in *P. exul* and *P.* aff. *exul*, while *trnQ-UUG* was duplicated in *P. charlesworthii*, *P. tigrinum*, and *P. barbigerum var. lockianum*. The two copies of *trnG-GCC* have one nucleotide variation, and the two copies of *trnQ-UUG* have eight nucleotide variations. Gene density ranged from 0.78 to 0.84, and *P. tigrinum* had the shortest plastome size and the highest gene density (Table S1). The GC content of the plastome genome ranged from 34.7% to 36.3%, and the GC content of the protein-coding genes ranged from 29.7% to 46.1%.

In addition, we found 17 genes containing introns, including six tRNA genes (*trnA-UGC*, *trnG-UCC*, *trnL-UAA*, *trnI-GAU*, *trnK-UUU*, and *trnV-UAC*) and 11 protein coding genes (*atpF*, *clpP*, *ndhB*, *petB*, *petD*, *rpl2*, *rpl16*, *rpoC1*, *rps12*, *rps16*, and *ycf3*). Eight of the protein coding genes contain one intron, while three of them (*clpP*, *rps12*, and *ycf3*) contain two introns (Table S2).

The LSC/IR_b_ boundary is relatively stable. While the LSC/IR_b_ junction is on *rpl22* in most species (76 of 81 samples), the LSC/IR_b_ junction is on *rps19* in *P. concolor* and *P. wenshenanse* × *P. bellatulum*, between *rpl22* and *rps19* in *P. rhizomatosum*, and between *rps19* and *trnH-GUG* in *P. hirsutissimum* (Fig. 4). Compared to the LSC/IR boundaries, the IR/SSC boundaries of *Paphiopedilum* varied among species (Fig. 4). Substantial variation in the SSC/IR boundary was mainly in subg. *Parvisepalum*. In most other samples (56 of 81 samples), one end of the SSC/IR junction was located in the intergeneric spacer region *trnL-ccsA*, near *trnL-UAG*, whereas the other junction of SSC/IR was located on the *ccsA* gene (Fig. 2).

The contraction of the SSC region resulted in the typical SSC genes being transferred to the IR region. One to six genes from the SSC region were transferred to the IR region. For example, *ycf1* was transferred to the IR region in all the sequenced samples, while Ψ*ndhD*, *psaC*, and *rps15* were incorporated into the IR region in most species. The gene *ccsA* expanded in the IR region occasionally, and *trnL-UAG* was transferred to the IR region in *P. delenatii*, *P. dianthum*, and *P. parishii* (Fig. 2).

The genomic comparison demonstrates that the SSC region of *Paphiopedilum* differs greatly in gene content, gene order, and gene orientation (Figs. 2 and 4, S2). The SSC regions of most species contain *trnL*, *rpl32*, and partial *ccsA*, while the SSC regions of five species are on the brink of losing, *P. appletonianum*, *P*. *barbigerum*, *P. emersonii*, *P. hirsutissimum*, and *P. villosum* only contain *trnL*-*UAG* in this region (Fig. 2). In addition, the genes *psaC* and Ψ*nadD* were preserved in the SSC region in six samples of subg. *Parvisepalum* (Fig. 2). In addition, there might be two copies of SSC with different directions in the same species [21]. Wang and Lanfear [22] used long-read sequencing to test the structural heteroplasmy in land plants and found the presence of chloroplast structural heteroplasmy in most land plant individuals, so the direction of the SSC region was not considered. Based on gene content and gene orientation, the SSC regions were classified into twelve types (Fig. S2), and type VIII is the dominant type (56 of 81 samples) (Fig. 2). Type I and type II are identical in gene content but differ in the gene direction of *rpl32* and *trnL-UAG* (Fig. S2). Type IX and type X are also identical in gene content, but in type IX, the two genes run in opposite directions, while in type X, the two genes run in the same direction (Fig. S2). Type XI and type XII both have *trnL-UAG*, but one nucleotide in type XII has shifted to the IR region. Subg. *Parvisepalum* has six types, whereas sect. *Cochlopetalum* has only one type (type VIII) (Fig. 2). When the SSC types are plotted on the phylogenetic tree, the result shows that the SSC types are not lineage-specific and that even the closely related species have distinct SSC types, such as species in subg. *Brachypetalum* and subg. *Parvisepalum* (Fig. 2). Surprisingly, the gene content of SSC regions has intraspecies variation. For example, one sample of *P. barbigerum* contains *trnL-UAG*, while the other sample contains *trnL-UAG* and *rpl32*. The two samples of *P. appletonianum* also have different SSC types (Fig. 2).

Gene gain and loss in *Paphiopedilum* samples was also analysed. Some of the gene losses are shared throughout the genus (e.g., some *ndh* genes), while other gene losses are lineage specific (Table S3). Most of the *ndh* genes were pseudogenized (Ψ*ndhD*, Ψ*ndhJ*, and Ψ*ndhK*) or lost (*ndhA*, *ndhC*, *ndhE*, *ndhF*, *ndhG*, *ndhH*, and *ndhI*) from the *Paphiopedilum* species plastome, except for *ndhB*. Most of the samples (76 of 81) sequenced in this study retained an intact copy of *ndhB*. In addition, the complete open reading frame of *ndhJ* was preserved in 23 samples, including four samples of sect. *Cochlopetalum* and 19 samples of sect. *Paphiopedilum* (Table S3). The genes *ndhC* and *ndhK* were preserved as pseudogenes in subg. *Parvisepalum* and sect. *Concoloria* but lost in the other species (Table S3). In particular, in five species sequenced (*P. barbatum*, *P. dayanum*, *P. platyphyllum*, *P. sugiyamanum*, and *P. tigrinum*), all 11 of the *ndh* genes were lost or pseudogenized (Fig. 2, Table S3).

In addition to the pseudogenes found in the *ndh* genes, we found that premature termination induced pseudogenization of other protein coding genes (*cemA* and *ycf15*). Most of the annotated copies (38 of 48) of *cemA* are preserved as pseudogenes, while all the annotated copies of *ycf15* were retained as pseudogenes (Table S3). The pseudogenization of *cemA* is mainly due to the slippage of poly structure and the appearance of premature stop codons, while the pseudogenization of *ycf15* is due to the appearance of more than one premature stop codon.

The plastomes of *Paphiopedilum* also show multiple structural rearrangements. We found widespread structural variation in the SSC regions, especially in subg. *Parvisepalum*, including the inversion and recombination of the SSC genes and the shift of the IR/SSC boundary (Figs. 2, 3 and 4). In particular, we found a 47 kb inversion spanning from *petN* to *clpP* in *P. fairrieanum*, which is absent in other species of *Paphiopedilum* (Figs. 3, S1).

### Phylogenetic analyses

Phylogenetic analyses were performed on the three concatenated datasets. The results of ML and BI analyses based on different matrices are almost identical except for the difference in support values, so we used the result based on the whole plastomes directly. Relationships among the sections are consistent with previous studies (Figs. 1 and 2, Fig. S3) [14, 15]. The phylogeny analyses showed subg. *Parvisepalum* at the base of the tree, followed by subg. *Brachypetalum*, and then the five sections in subg. *Paphiopedilum*. The five sections grouped into two clades, with one clade formed by sect. *Cochlopetalum-*(sect. *Coryopedilum*, sect. *Pardlopetalum*) and the other clade formed by sect. *Barbata* and sect. *Paphiopedilum*, with just a few terminal nodes in the tree are still unresolved, especially species in sect. *Paphiopedilum* (Fig. S3 and S4).

Additionally, the four enigmatic species of *Paphiopedilum* all nested in subg. *Paphiopedilum*. *P. fairrieanum* clustered with the sect. *Paphiopedilum*-sect. *Barbata* clade with a high support value (BP = 100, PP = 1.00), and *P. canhii* clustered with the sect. *Cochlopetalum -*(sect. *Coryopedilum*, sect. *Pardlopetalum*) clade (BP = 94, PP = 1.00); meanwhile, *P. rungsuriyanum* and *P. hirsutissimum* formed a clade with a high support value (BP = 91, PP = 1.00), and the relationship with other branches is unresolved (Fig. S3). Removing the four taxa results in a tree with the same topology but with elevated support values for some of the branches (Fig. S4).

### Nucleotide substitution rate analyses

**Fig. 5:**
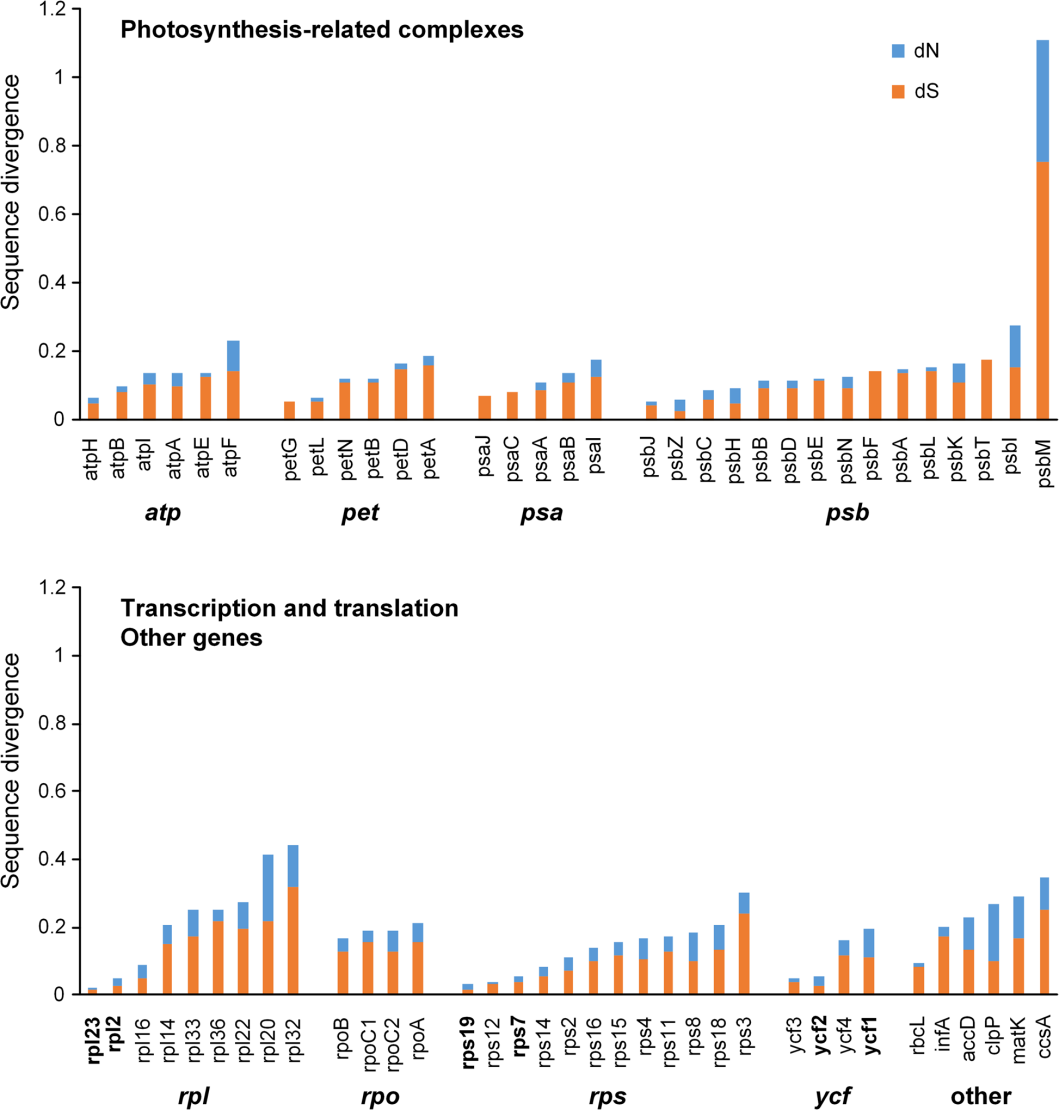
Synonymous (dN) and nonsynonymous (dS) substitution rates of *Paphiopedilum* chloroplast genes grouped by complex and function. Gene names blackened are genes in the IR region

Mean synonymous and nonsynonymous divergence was low (dN = 0.0471, dS = 0.1222) with the dS value almost three times that of dN (Fig. 5, Table S4). We detected signals of positive selection in *clpP* (dN/dS = 1.6561), *psbH* (dN/dS = 1.0318), and *psbZ* (dN/dS = 1.5768). Our analyses show that both dN and dS had significantly increased in *psbM* (dN = 0.3585, dS = 0.7519) (Fig. 5), which is induced by a frameshift mutation in *P. niveum* (KJ524105). Genes in the SC regions have higher substitution rates than genes in the IR regions. Unexpectedly, genes transferred to the IR region due to boundary shift did not show lower substitution rates (Fig. 5, Table S4).

## Discussion

### The phylogeny of *Paphiopedilum*

Our phylogeny based on the whole plastome resolves the relationship among sections, which is largely consistent with the most recent phylogenies obtained from partial cytoplasmic DNA markers (Fig. S3) [14, 15]. The phylogeny resolved the monophyly of sect. *Cochlopetalum* with high bootstrap support (BP = 98, PP = 1.00), while this clade was poorly resolved in previous studies [14, 15]. The four enigmatic species all fall outside the established sections. *P. fairrieanum* clusters with sect. *Paphiopedilum*-sect. *Barbata* clade with a high support value (BP = 100, PP = 1.00), *P. canhii* clusters with sect. *Cochlopetalum-*(sect. *Coryopedilum*, sect. *Pardlopetalum*) clade with a medium support value (BP = 94, PP = 1.00) (Fig. S3). However, the phylogenetic position of the other two enigmatic taxa (*P. hirsutissimum* and *P. rungiyasanum*) clustered together with a high support value (BP = 99, PP = 1.00) and formed parallel relationships with *P. fairrieanum*, sect. *Paphiopedilum*, and sect. *Barbata* (Fig. S3). Notably, the support values of several clades slightly increased after the removal of these species. For example the support value of sect. *Cochlopetalum* increased to 99 (Fig. S4). The uncertain phylogenetic positions of the enigmatic taxa might be related to the heteroplasmy found in the chloroplast genomes (unpublished data). The short branch lengths in sect. *Paphiopedilum* suggest their recent radiation, which means species in this section are in their preliminary stage of speciation. On the other hand, the long branch length represents the differentiation from the closely related species. For example, the long branch of *P. druryi* might be due to its isolated distribution in south India.

### The chloroplast genome evolution of *Paphiopedilum*

The largest (*P. emersonii*, 164,092 bp) and smallest (*P. tigrinum*, 152,130 bp) plastome sizes differ by ~ 12 kb, and the gene number ranges from 127 to 134 genes (Table S1). The genome size and gene number variation are mainly due to the IR expansion and the loss of the *ndh* genes, and the genome size is larger than the average plastome size (151 kb) [6]. Compared with other sequenced chloroplast genomes of photoautotrophic orchid, such as *Dendrobium* [23] and *Holcoglossum* [24], the plastomes of *Paphiopedilum* contain more size variation and structure variation at the genus level (Fig. 1, Table S1). In addition, the GC content of the genus (34.7 − 36.3%) is also more variable and lower at the genus level than in other sequenced genera in Orchidaceae, e.g., *Cymbidium* (36.8 − 37%) [25], *Dendrobium* (37.31 − 37.68%) [23], and *Holcoglossum* (35.3 − 35.5%) [24]. The lower GC content might relate to the extremely lengthy AT-repeat regions in the genus. We found AT repeats more than 100 bp long in some species, especially in the 11 samples with gaps in the LSC regions (Table S1).

The LSC/IR_b_ boundary of *Paphiopedilum* is relatively stable; LSC/IR_b_ resided on *rpl22* in most species, while the IR/SSC boundary of the genus is different from those in other species of orchids and variable at the genus level (Fig. 4). Owing to the instability of the IR/SSC boundaries, the chloroplast genome of *Paphiopedilum* experienced IR expansion and SSC contraction. The typical land plant IR is 25 kb [6, 7], while the IR region of *Paphiopedilum* is 32 – 37 kb, and the IRs account for approximately 40 – 45% of the plastomes. The increased length in the IR regions results from the IR/SSC boundary shifts.

A large IR expansion (several kilobases) has also been reported in other angiosperm lineages, including *Acacia* and *Inga* [26], *Erodium* [27], *Passiflora* [28, 29], and *Pelargonium* [8, 30]. In *Pelargonium*, IR expanded towards both the LSC and the SSC regions. However, the IR expansions in *Paphiopedilum* mainly included former SSC genes. IR expansion into the SSC region has also been reported in other species, e.g., *Musa acuminate* [31], *Pedicularis ishidoyana* [32], and *Vanilla* [11, 13, 33].

The large IRs of the plastomes are hypothesized to contribute to plastome stabilization because their absence often coincides with severe changes in gene order [34]. IRs are thought to stabilize the plastome through homologous recombination-induced repair mechanisms [35]. However, large IRs have no impact on the plastome stability of the genus. With the shifts in the IR/SSC boundaries, the SSC regions in *Paphiopedilum* have shortened and variable SSC lengths (524 – 5913 bp). The tightening of the SSC regions is associated with the loss of *ndh* genes and the transfer of former SSC genes to the IR region. For example, the 524 bp SSC region of *P. hirsutissimum* is smaller than other sequenced plastomes in Orchidaceae. The strong shrinking of SSC regions was also documented in other unlinked photoautotrophic plants, such as *Annona cherimola* (2966 bp) [36], *Asarum* (0 – 14 bp) [37], *Lamprocapnos spectabilis* (1645 bp) [38], *P. ishidoyana* (27 bp) [32], *Pelargonium* (c. 6.7 kb) [8], and *Vanilla* (c. 2 kb) [11, 13, 33]. Interestingly, the other genera that experienced SSC contraction have a relatively stable SSC length at the genus level, e.g., all the sequenced plastomes of *Pelargonium* shared a 6.7 kb SSC region [8], while the length of the SSC region of *Paphiopedilum* is more variable. The SSC-contracted plastomes of *Paphiopedilum* have unique features compared with other tightened SSC lineages, including gene content variation, length variation, and gene rearrangement (Figs. 2, 3 and 4), according to the sequenced chloroplast genomes of other genera of slipper orchids [10, 11, 39], suggesting that SSC contraction occurred after *Paphiopedilum* split from its sister clade.

In addition to size variation, the SSC region of *Paphiopedilum* experienced extensive gene rearrangement (Fig. 3). The SSC regions of most species in sect. *Concoloria*, sect. *Cochlopetalum*, sect. *Coryopedilum*, sect. *Paphiopedilum*, and sect. *Barbata* retain *trnL-UAG*, *rpl32* and a truncated *ccsA* fragment (*trnL-UAG*( +)*-rpl32*( +)*-ccsA*-P( +), type VIII, approximately 1.8 kb). In the remaining species, the gene content, gene order, and length of the region are highly variable (524 – 5913 bp) (Figs. 1 and 2, S2; Table S1). In other angiosperm, there are two gene clusters in the SSC region, namely*, rpl32*( +)-*trnL-UAG*( +)-*ccsA*( +) and *ndhD*( −)-*psaC*( −)-*rps15*( −). In other orchids, such as Apostasioideae [40], *Cypripedium* [9, 39], *Cymbidium* [25]*,* and *Phalaenopsis aphrodite* [41], the genes on the two clusters are in opposite orientations. However, the original *rpl32*( +)-*trnL-UAG*( +)-*ccsA* ( +) linkage was only preserved in *P. armeniacum* [10]. In other species of the genus, the above gene linkages were broken and changed to *trnL-UAG*( +)-*rpl32*( +)-*ccsA*( +) and *trnL-UAG*( −)-*rpl32*( −)-*ccsA*( −) in most cases, which means that there is inversion and recombination in the SSC region. Gene arrangement brought *rpl32* and *ccsA* (which are normally separated in the plastome) next to each other (Figs. 2 and 4). In addition, we found a 47 kb inversion in the LSC region of *P. fairieanum* (Fig. 3, S1c). A 61 kb inversion in the LSC region was previously reported in *Cypripedium formosanum* [11]. But the mechanisms for these inversions are unknown.

Complete gene duplications (of *rpl32*, *trnL-UAG*, *ccsA*, Ψ*ndhD*, *psaC*, *rps15*, and *ycf1*) were documented due to the expansion of the IR region. Additionally, gene duplications outside of the IR regions was also documented. We found that *trnG-GCC* was duplicated in the LSC in two species, while *trnQ-UUG* was duplicated in the LSC in three species, and the two duplication events both occurred in sect. *Paphiopedilum*. The second copies of *trnG-GCC* and *trnQ-UUG* are identical to sequences from other orchids, indicating that the two copies might be horizontally transferred, especially *trnQ-UUG*. The two copies of the gene have eight nucleotide variations. The duplication of *trnQ-UUG* has also been documented in other studies [42, 43].

Most of the *ndh* genes were pseudogenized or lost from the genus, but the functional *ndhJ* and *ndhB* were preserved in some lineages (Table S3). Considering that *ndhA*, *ndhE*, *ndhF*, *ndhG*, *ndhH*, and *ndhI* were also lost in *P. longifolium* [10] whereas they were preserved in *C. formosanum* [11] and *C. japonicum* [39], we infer that the loss of these genes occurred in the ancestors of the conduplicate-leaved genera. The pseudo copies of *ndhK* and *ndhC* were preserved in most samples of subg. *Parvisepalum* and subg. *Brachypetalum* (Table S3) and lost in subg. *Paphiopeilum*, suggesting that *ndhK* and *ndhC* were lost in the ancestor of subg. *Paphiopeilum*. Considering the retention of *ndhJ* and *ndhB* in the genus (Table S3), we infer that the loss/pseudogenization of *ndhB* and *ndhJ* probably occurred rather recently because they are repeatedly retained in the terminal taxa.

Furthermore, four of the five species with all 11 *ndh* genes pseudogenized or lost are distributed on islands, and the other one (*P. tigrinum*) is distributed on the mainland. The five species are all photoautotrophic plants. In addition, the five species belong to three sections (Fig. 2) and are nested in different clades, suggesting that there are four independent *ndh* loss/pseudogenization events at the genus level. The above evidence provided clues that the *ndh* complex is undergoing degradation in this genus, and the degradation of the *ndh* genes is not lineage specific, suggesting a very recent pseudogenization of some *ndh* genes. The independent degradation of *ndh* genes was also found in *Cymbidium* [44]. In addition, the loss of the full set of plastid *ndh* genes in other photoautotrophic orchids, such as *Erycina pusilla* [45], *Holcoglossum* [24], *P. aphrodite* [41], and *Vanilla planifolia* [11], has also been reported in previous studies*.* Lin et al. [46] even proposed that the loss of *ndh* complexes probably increased the transition of the life history from photoautotrophic to heterotrophic.

Kim et al. [8] proposed that the *ndhF* gene strongly correlated with the IR/SSC junction stability, whereas the result of Niu et al.’s [13] study indicated that the *ndh* genes strongly related to the IR/SSC junction stability. Though the IR/SSC boundary is different from other orchids but relatively stable in most *Paphiopedilum* species, we infer that *ndhF* or *ndh* is not correlated with the IR/SSC junction stability at the genus level. The *ndhF* gene was lost in all the sequenced species of *Paphiopedilum*, and the five species that lost all 11 functional *ndh* genes have stable IR/SSC junctions (Fig. 2). We infer that there are other underlying mechanisms related to the structure variations in the genus.

### The substitution rates of the chloroplast genes

The substitution rate of the genus is low (dN = 0.0471, dS = 0.1222) and varied among genes, with the dS value almost three times that of dN (Fig. 5, Table S4). We detected signals of positive selection in *clpP* (dN/dS = 1.6561), *psbH* (dN/dS = 1.0318), and *psbZ* (dN/dS = 1.5768). In several angiosperm lineages, such as *Acacia* and *Inga* [26], *Passiflora* [29], and *Silene* [47], positive selection of *clpP*, which is involved in protein metabolism, has been identified. Two other positively selected genes are involved in photosynthesis: *psbH* was under positive selection in the shade tolerant *Oryza* [48], and psbZ showed positive selection in *Rhododendron pulchrum* [49].

Previous studies have revealed that genes in the IR region have lower substitution rates than genes in the SC region [30, 50, 51]. Genes in the IR region also have relatively lower substitution rates compared to SC region genes in orchids [25]. Unexpectedly, *ycf1* moved to the IR region but still had a high substitution rate (dN = 0.0821, dS = 0.1140), and its substitution rate is higher than that the other genes in the IR region. Even *psaC* (dN = 0, dS = 0.0825) and *rps15* (dN = 0.0388, dS = 0.1183), which transferred to the IR region, have higher divergence than the other genes in the IR region. Furthermore, *ccsA* (dN = 0.0932, dS = 0.2543) and *rpl32* (dN = 0.1222, dS = 0.3193) were mostly retained in the SSC region and have higher divergence than other genes in the SSC region (Fig. 5). Weng et al. [8] found similar phenomena in *Pelargonium*, where the expanded IR genes in plastid genomes did not have lower substitution rates. Interestingly, the divergence of photosynthesis-related genes is relatively lower than the divergence of other genes (Fig. 5).

## Conclusion

Analysing the chloroplast genome is a key way to study the molecular evolution of orchids. However, previously published orchid studies mainly focused on heterotrophic species and the subfamily Epidendroideae. We used comparative chloroplast genome sequences to reveal the evolutionary history of *Paphiopedilum*. The genome size of *Paphiopedilum* is slightly larger than that of other angiosperm, and all the species in *Paphiopedilum* experienced IR expansion and SSC contraction. The expansion of the IR region ranged from ~ 7 kb to ~ 12 kb, and the IR expansion in *Paphiopedilum* is associated with the transfer of former SSC genes to the IR region. The comparison of plastomes showed that IR expansion and *ndh* loss contributed to the size variation in the genus. Because of the variation in the IR/SSC boundaries, the SSC region of *Paphiopedilum* chloroplast genomes showed massive variation in length and gene content. *Paphiopedilum* provides the best opportunity to study the dynamics of chloroplast genome evolution and to test the rate of heterogeneity related to the variation in the IR/SSC boundaries. Though genome rearrangement is mainly reported in the mitochondrial genome (e.g. [52]), this study provides evidence of highly active genome rearrangements in the SSC regions of *Paphiopedilum* with dense sampling. The *ndh* genes of *Paphiopedilum* experienced varying degrees of degradation, and five species have lost/pseudogenized all 11 *ndh* genes. However, the mechanisms underlying the loss/pseudogenization of these genes are unknown. This study sheds light on the tempo and mode of evolutionary changes that occurred in the chloroplast genomes across *Paphiopedilum* and provides a good example of how to study the chloroplast genome evolution of Orchidaceae.

## Methods

### Taxon sampling and library construction

Plant materials sequenced in this study were collected from the National Orchid Conservation & Research Center of Shenzhen (NOCC) (Table S1), including four species downloaded from GenBank. A total of 81 samples representing 63 species and covering seven major clades of *Paphiopedilum*, four natural hybrids and one man-made hybrid were also included. Based on previous phylogenetic studies, *P. longifolium* was chosen as the outgroup.

Total genomic DNA was extracted from fresh leaves using the CTAB method [53]. Paired-end libraries with 350 bp inserts were constructed for this study, and sequencing was performed at Novogene (Beijing, China) on the Hiseq platform (150 bp). And we obtained 20 G of raw data for each sample.

### Sequence assembling and annotation

High-quality filtered paired-end reads were filtered in CLC Genomics Workbench v.10.1.1 (https://www.qiagenbioinformatics.com/) reference to published plastomes, and then de novo assembled using CLC Genomics Workbench v.10.1.1 (with different word sizes and bubble sizes) or NOVOPlasty3.5 (K-mer = 39, *rbcL* from *P. armeniacum* was used as the seed sequence) [54]. The assembled contigs were merged in Geneious v.11.1.2 (Biomatters, Inc.) to build draft plastomes. We then mapped reads to draft plastomes in Geneious v.11.1.2 to check the ambiguous regions. Annotation of the plastomes was performed in Geneious v.11.1.2 (Biomatters, Inc.) coupled with manual corrections.

Plastome maps were generated with OrganellarGenomeDRAW [55]. The boundaries of IR and SC regions were defined by REPuter [56] with default settings. We calculated GC content in Geneious v.11.1.2. Then, changes in genome structure were visualized using progressive Mauve [57] with IRa removed. Protein coding regions (CDS), rRNA, tRNA, intergenic spacers (IGS), and intron sequences were extracted and aligned to generate original CDS, rRNA, tRNA, IGS, and intron alignments.

### Phylogenetic analysis

For phylogenetic analyses, 68 protein-coding gene sequences (*ndhB* included), four rRNA gene sequences, 30 tRNA gene sequences, and 87 intergenic spacer regions (Tables S2, S5) were aligned with Muscle v3.8.1551 [58] with default settings and refined manually. The poorly aligned regions of IGS and introns were removed with Gblocks v 0.91b [59] with default settings, except with gaps allowed in up to half of sequences. Then, they were concatenated into different datasets. We obtained three concatenated datasets: 1) protein-coding sequences (CDS) and rDNA; 2) intergenic spacer (IGS), intron, and tRNA; and 3) whole plastomes (CDS + IGS + intron + rRNA + tRNA).

Maximum likelihood (ML) trees were inferred from RAXml 8.2.4 [60] under the GTRGAMMA model with 1000 bootstrap replicates. Bayesian inferences (BI) were performed with MrBayes [61]. PartitionFinder v2.1.1 [62] was used to determine the optimal partitioning schemes with pre-defined partitioning by genes/intergenic-spacers under corrected Akaike Information Criterion (AICc) and greedy search. The partitioning schemes were used in the following analyses. For the Bayesian inference, one cold and three incrementally heated Markov chain Monte Carlo (MCMC) chains were run for 10,000,000 cycles and repeated twice to avoid spurious results. One tree per 1000 generations was sampled, with a burn-in of the first 2500 samples for each run.

### Nucleotide substitution rate analyses

We used the CODEML programme in PAML v. 4.9 (model = 0) [63] to calculate the average nonsynonymous substitution rate (dN) and synonymous substitution rate (dS) for 67 protein coding genes by the F3X4 codon model. Gapped regions were excluded for rate estimation (cleandata = 1). The ML tree inferred from whole plastome was used as the input tree.

### Correlation analysis

Correlation between IR length and SCC length were calculated in RStudio version 1.4.1106 [64]. The cor.test function was used for assessing *p*-values with Pearson’s correlation test.

## Supplementary Information

**Additional file 1: Figure S1.** Chloroplast genome structure of *Paphiopedilum*. a) *Paphiopedilum charlesworthii*, b) *P. emersonii*, c) *P. fairrieanum*, and d) *P. vietnamense*. **Figure S2.** The twelve SSC types found in *Paphiopedilum*. **Figure S3.** Phylogenetic tree (ML) of *Paphiopedilum* based on whole plastomes. The number above the branches are the bootstrap values ≥ 70 and Bayesian posterior probabilities ≥ 0.90. The branched in bold are the four unstable species. **Figure S4.** Phylogenetic tree (ML) of *Paphiopedilum* based on whole plastomes with four unstable species excluded. The number above the branches are the bootstrap values ≥ 70 and Bayesian posterior probabilities ≥ 0.90.

**Additional file 2: Table S1.** Details of accessions included in this study. **Table S2.** List of genes identified in the chloroplast genomes of *Paphiopedilum*. **Table S3.** Gene losses and pseudogenes in *Paphiopedilum*. **Table S4.** Non-synonymous substitution rate (dN), synonymous substitution rate (dS), and dN/dS for each gene. **Table S5.** List of intergenic spacer regions used in this study.

## Data Availability

All plastomes generated in this study are deposited in NCBI database (https://www.ncbi.nlm.nih.gov/) (GenBank accession Nos. MN587749 – MN587825, see Table S1), and all the datasets supported the conclusion are available at Dryad Digital Repository. These data will remain private until the related manuscript has been accepted. All other data generated in this manuscript are available from the corresponding author upon reasonable request.

## Abbreviations

AIC: Akaike information criterion
AT: Adenosine Thymine
BI: Bayesian inference
BP: Bootstrap probability
bp: Base pair
BS: Branch support
CDS: Protein-coding sequences
cp: Chloroplast
CTAB: Cetyltrimethylammonium bromide
dN: Non-synonymous
dS: Synonymous mutation
GC: Guanine-cytosine
GTR: General time reversible
IGS: Intergenic sequences
IR: Inverted repeat
IR_a_: Inverted repeat A
IR_b_: Inverted repeat B
IRs: Inverted repeat regions
kb: Kilobase
LSC: Large single copy
ML: Maximum likelihood
rRNAs: Ribosomal RNAs
SC: Single copy
SSC: Small single copy
tRNAs: Transfer RNAs
