# Supplementary material for: The chloroplast genome evolution of Venus slipper (Paphiopedilum): IR expansion, SSC contraction, and highly rearranged SSC regions
Source: BMC Plant Biol. 2021 May 31;21:248. doi: 10.1186/s12870-021-03053-y (PMC8165784; doi:10.1186/s12870-021-03053-y)
Supplement: Supplementary file 1 — Additional file 1: Figure S1. Chloroplast genome structure of Paphiopedilum. a) Paphiopedilum charlesworthii, b) P. emersonii, c) P. fairrieanum, and d) P. vietnamense. Figure S2. The twelve SSC types found in Paphiopedilum. Figure S3. Phylogenetic tree (ML) of Paphiopedilum based on whole plastomes. The number above the branches are the bootstrap values ≥ 70 and Bayesian posterior probabilities ≥ 0.90. The branched in bold are the four unstable species. Figure S4. Phylogenetic tree (ML) of Paphiopedilum based on whole plastomes with four unstable species excluded. The number above the branches are the bootstrap values ≥ 70 and Bayesian posterior probabilities ≥ 0.90. [file 12870_2021_3053_MOESM1_ESM.pdf]

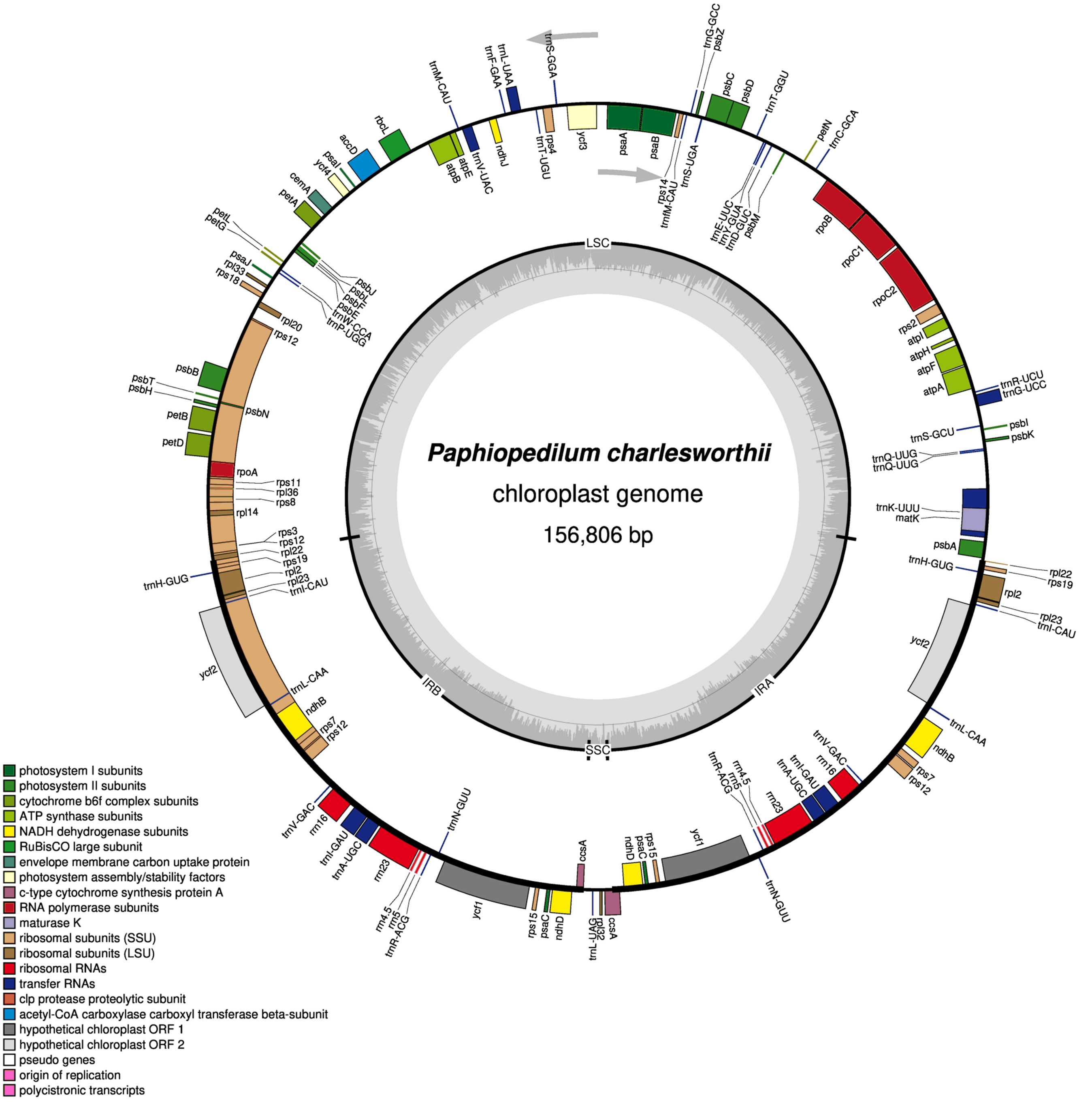

Paphiopedilum charlesworthii
chloroplast genome
156,806 bp
LSC
IRA
IRB
SSC
trnG-GCC
psbZ
psbC
psbD
trnT-GGU
petN
trnC-GCA
trnS-GGA
trnL-UAA
trnF-GAA
trnM-CAU
rbcL
accD
psaI
ycf4
cemA
petA
petL
petG
psaJ
rpl33
rps18
rpl20
rps12
psbB
psbT
psbH
petB
petD
psbN
rpoA
rps11
rpl36
rps8
rpl14
rps3
rps12
rpl22
rps19
rpl2
rpl23
trnI-CAU
trnH-GUG
ycf2
trnL-CAA
ndhB
rps7
rps12
atpE
atpB
trnV-UAC
ndhJ
rps4
trnT-UGU
ycf3
psaA
psaB
rps14
trnfM-CAU
trnS-UGA
trnE-UUC
trnY-GUA
trnD-GUC
psbM
rpoB
rpoC1
rpoC2
rps2
atpI
atpH
atpF
atpA
trnR-UCU
trnG-UCC
psbI
psbK
trnS-GCU
trnQ-UUG
trnQ-UUG
trnK-UUU
matK
psbA
trnH-GUG
rpl22
rps19
rpl2
rpl23
trnI-CAU
ycf2
trnL-CAA
ndhB
rps7
rps12
trnV-GAC
rrn16
trnI-GAU
trnA-UGC
rrn23
rrn4.5
rrn5
trnR-ACG
trnN-GUU
ycf1
rps15
psaC
ndhD
ccsA
trnL-UAG
rpl32
ccsA
ndhD
psaC
rps15
ycf1
trnN-GUU
rrn23
trnR-ACG
rrn4.5
rrn5
trnA-UGC
trnI-GAU
rrn16
trnV-GAC
psbJ
psbL
psbF
psbE
trnW-CCA
trnP-UGG
photosystem I subunits
photosystem II subunits
cytochrome b6f complex subunits
ATP synthase subunits
NADH dehydrogenase subunits
RuBisCO large subunit
envelope membrane carbon uptake protein
photosystem assembly/stability factors
c-type cytochrome synthesis protein A
RNA polymerase subunits
maturase K
ribosomal subunits (SSU)
ribosomal subunits (LSU)
ribosomal RNAs
transfer RNAs
clp protease proteolytic subunit
acetyl-CoA carboxylase carboxyl transferase beta-subunit
hypothetical chloroplast ORF 1
hypothetical chloroplast ORF 2
pseudo genes
origin of replication
polycistronic transcripts

Fig. S1a

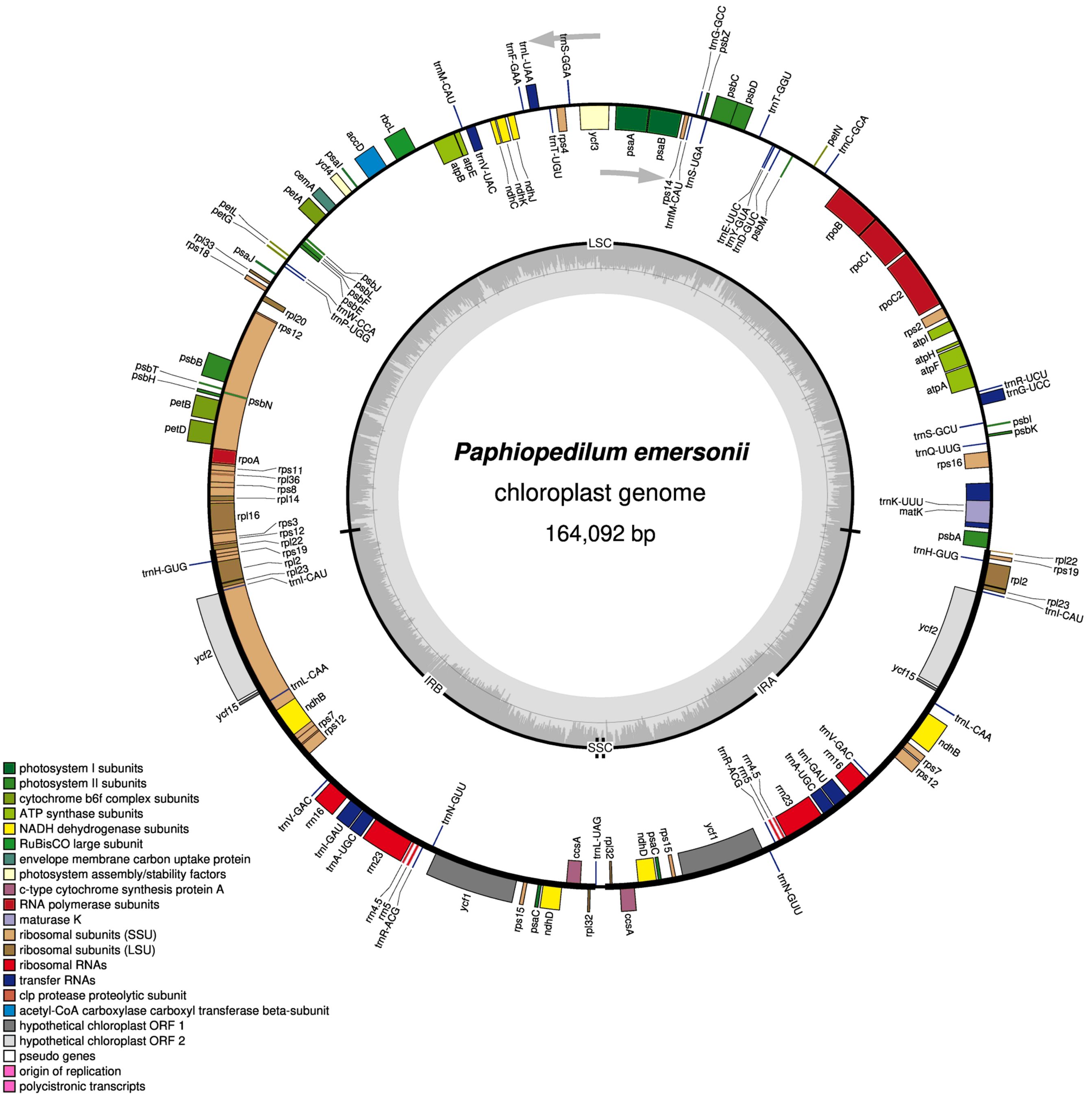

Paphiopedilum emersonii
chloroplast genome
164,092 bp
LSC
IRA
IRB
SSC
trnG-GCC
psbZ
psbC
psbD
trnT-GGU
petN
trnC-GCA
rpoB
rpoC1
rpoC2
rps2
atpI
atpH
atpF
atpA
trnR-UCU
trnG-UCC
psbI
psbK
trnS-GCU
trnQ-UUG
rps16
trnK-UUU
matK
psbA
trnH-GUG
rpl22
rps19
rpl2
rpl23
trnI-CAU
ycf2
ycf15
trnL-CAA
ndhB
rps7
rps12
trnV-GAC
rrn16
trnI-GAU
trnA-UGC
rrn23
rrn4.5
rrn5
trnR-ACG
trnN-GUU
ycf1
rps15
psaC
ndhD
rpl32
trnL-UAG
ccsA
rpoA
rps11
rpl36
rps8
rpl14
rpl16
rps3
rpl20
psbB
psbT
psbH
psbN
petB
petD
rps18
rpl33
psaJ
petL
petG
psbJ
psbL
psbF
psbE
trnW-CCA
trnP-UGG
petA
cemA
ycf4
psaI
accD
rbcL
atpE
atpB
trnV-UAC
trnM-CAU
trnF-GAA
trnL-UAA
trnS-GGA
trnT-UGU
rps4
ycf3
ndhJ
ndhK
ndhC
psaA
psaB
rps14
trnfM-CAU
trnS-UGA
trnE-UUC
trnY-GUA
trnD-GUC
psbM
photosystem I subunits
photosystem II subunits
cytochrome b6f complex subunits
ATP synthase subunits
NADH dehydrogenase subunits
RuBisCO large subunit
envelope membrane carbon uptake protein
photosystem assembly/stability factors
c-type cytochrome synthesis protein A
RNA polymerase subunits
maturase K
ribosomal subunits (SSU)
ribosomal subunits (LSU)
ribosomal RNAs
transfer RNAs
clp protease proteolytic subunit
acetyl-CoA carboxylase carboxyl transferase beta-subunit
hypothetical chloroplast ORF 1
hypothetical chloroplast ORF 2
pseudo genes
origin of replication
polycistronic transcripts

Fig. S1b

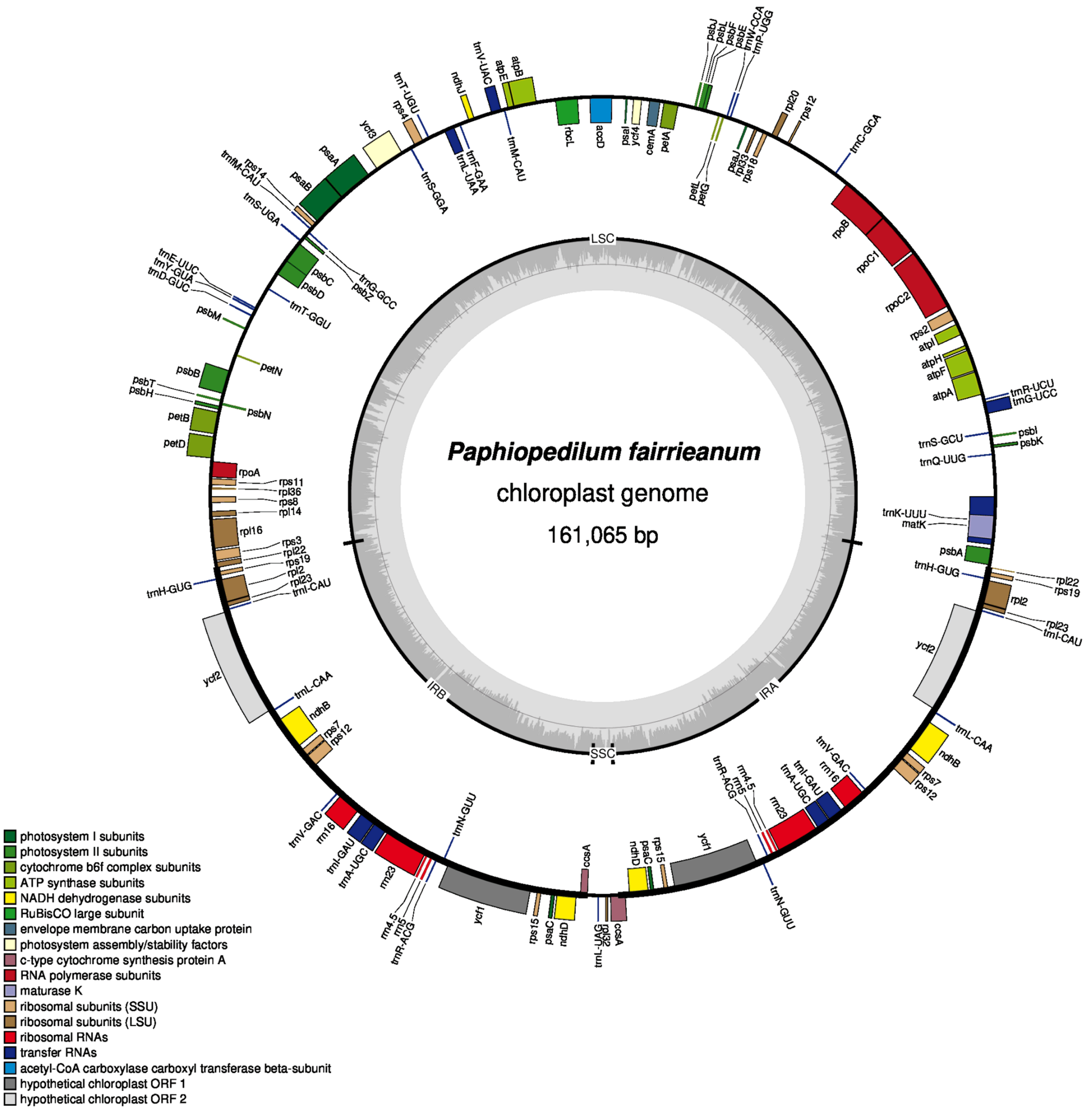

Paphiopedilum fairrieanum
chloroplast genome
161,065 bp
LSC
SSC
IRA
IRB
photosystem I subunits
photosystem II subunits
cytochrome b6f complex subunits
ATP synthase subunits
NADH dehydrogenase subunits
RuBisCO large subunit
envelope membrane carbon uptake protein
photosystem assembly/stability factors
c-type cytochrome synthesis protein A
RNA polymerase subunits
maturase K
ribosomal subunits (SSU)
ribosomal subunits (LSU)
ribosomal RNAs
transfer RNAs
acetyl-CoA carboxylase carboxyl transferase beta-subunit
hypothetical chloroplast ORF 1
hypothetical chloroplast ORF 2
psbJ
psbL
psbF
psbE
trnW-CCA
trnP-UGG
rpl20
rps12
trnC-GCA
rpoB
rpoC1
rpoC2
rps2
atpI
atpH
atpF
atpA
trnR-UCU
trnG-UCC
psbI
psbK
trnS-GCU
trnQ-UUG
trnK-UUU
matK
psbA
trnH-GUG
rpl22
rps19
rpl2
rpl23
trnI-CAU
ycf2
trnL-CAA
ndhB
rps7
rps12
trnV-GAC
rrn16
trnI-GAU
trnA-UGC
rrn23
rrn4.5
rrn5
trnR-ACG
trnN-GUU
ycf1
rps15
psaC
ndhD
ccsA
trnL-UAG
rpl32
rbcL
accD
psaI
ycf4
cemA
petA
petL
petG
psaJ
rpl33
rps18
atpB
atpE
trnV-UAC
ndhJ
trnT-UGU
rps4
ycf3
psaA
psaB
rps14
trnfM-CAU
trnS-UGA
trnE-UUC
trnY-GUA
trnD-GUC
psbM
petN
psbB
psbT
psbH
psbN
petB
petD
rpoA
rps11
rpl36
rps8
rpl14
rpl16
rps3
trnM-CAU
trnF-GAA
trnL-UAA
trnS-GGA
psbC
psbD
psbZ
trnG-GCC
trnT-GGU

Fig. S1c

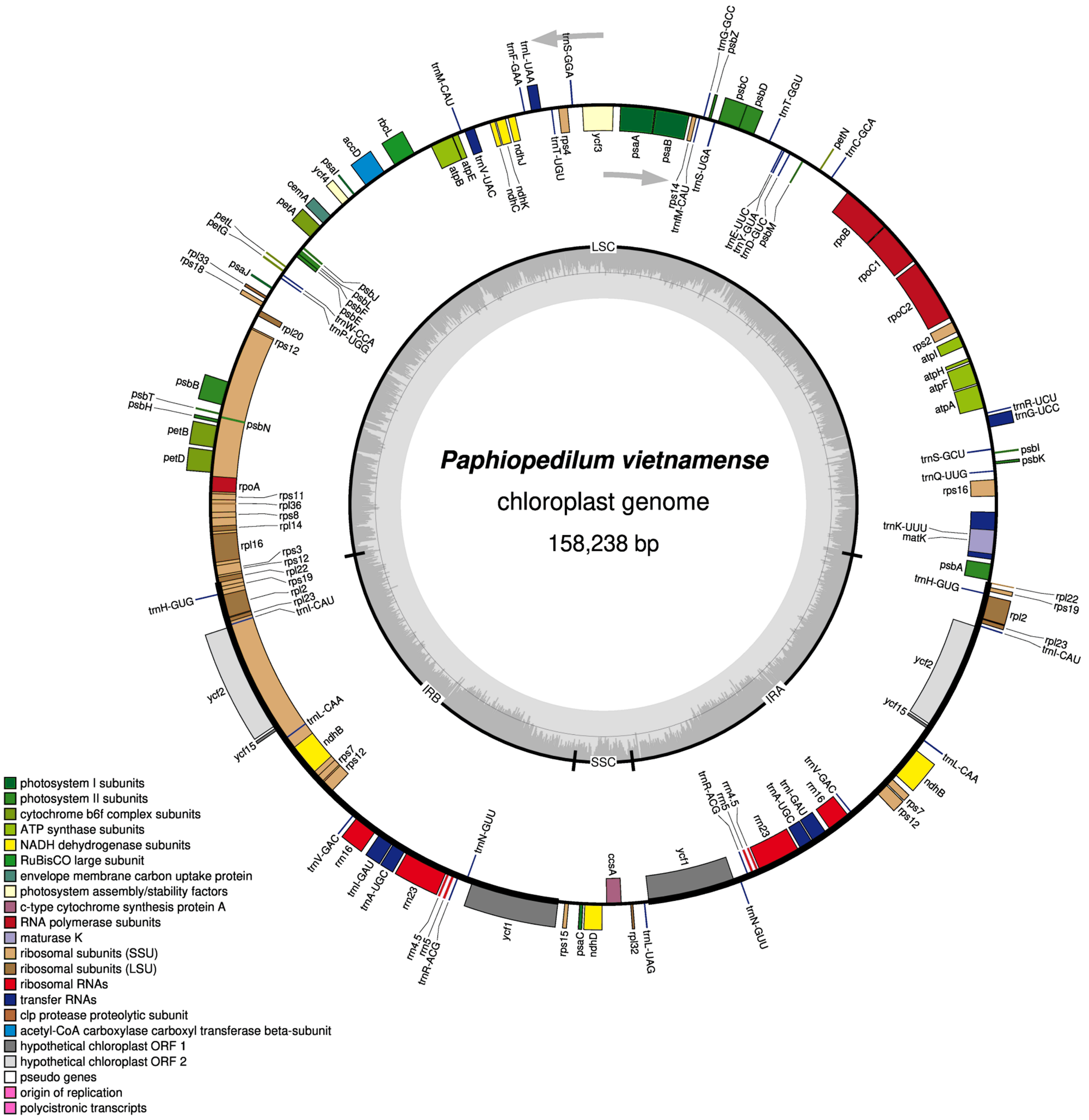

Fig. S1d

Fig. S1. Chloroplast genome structure of *Paphiopedilum*. a) *Paphiopedilum charlesworthii*, b) *P. emersonii*, c) *P. fairrieanum*, and d) *P. vietnamense*.

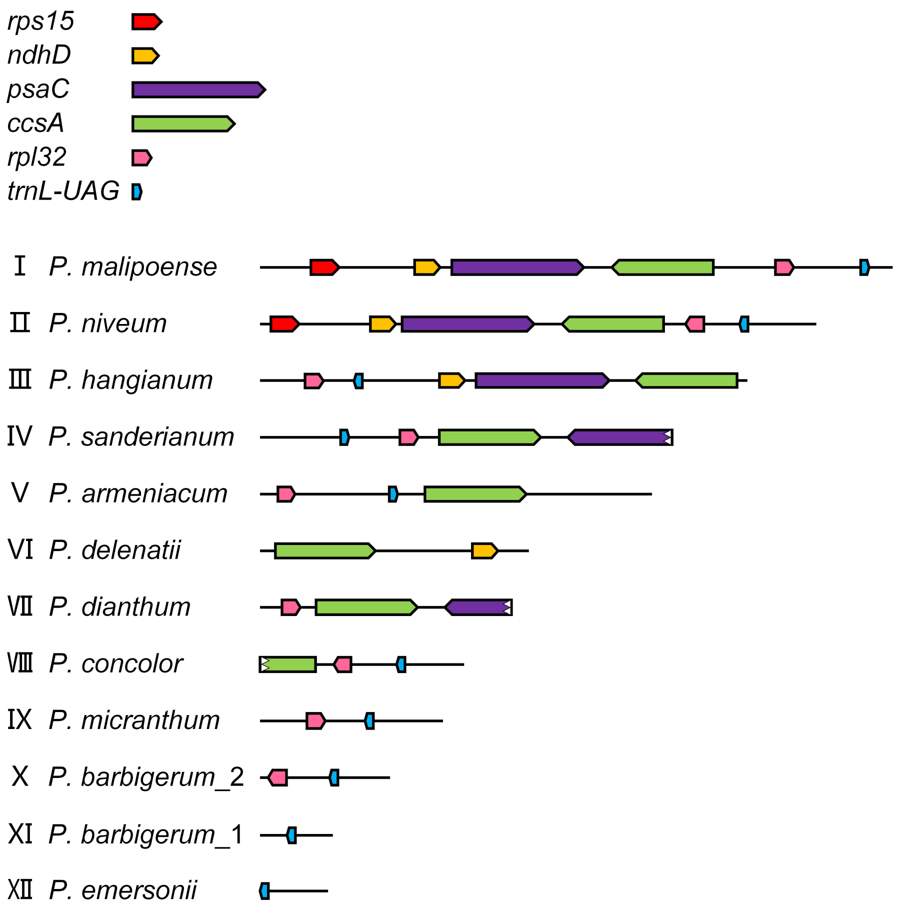

Fig. S2. The twelve SSC types found in *Paphiopedilum*.

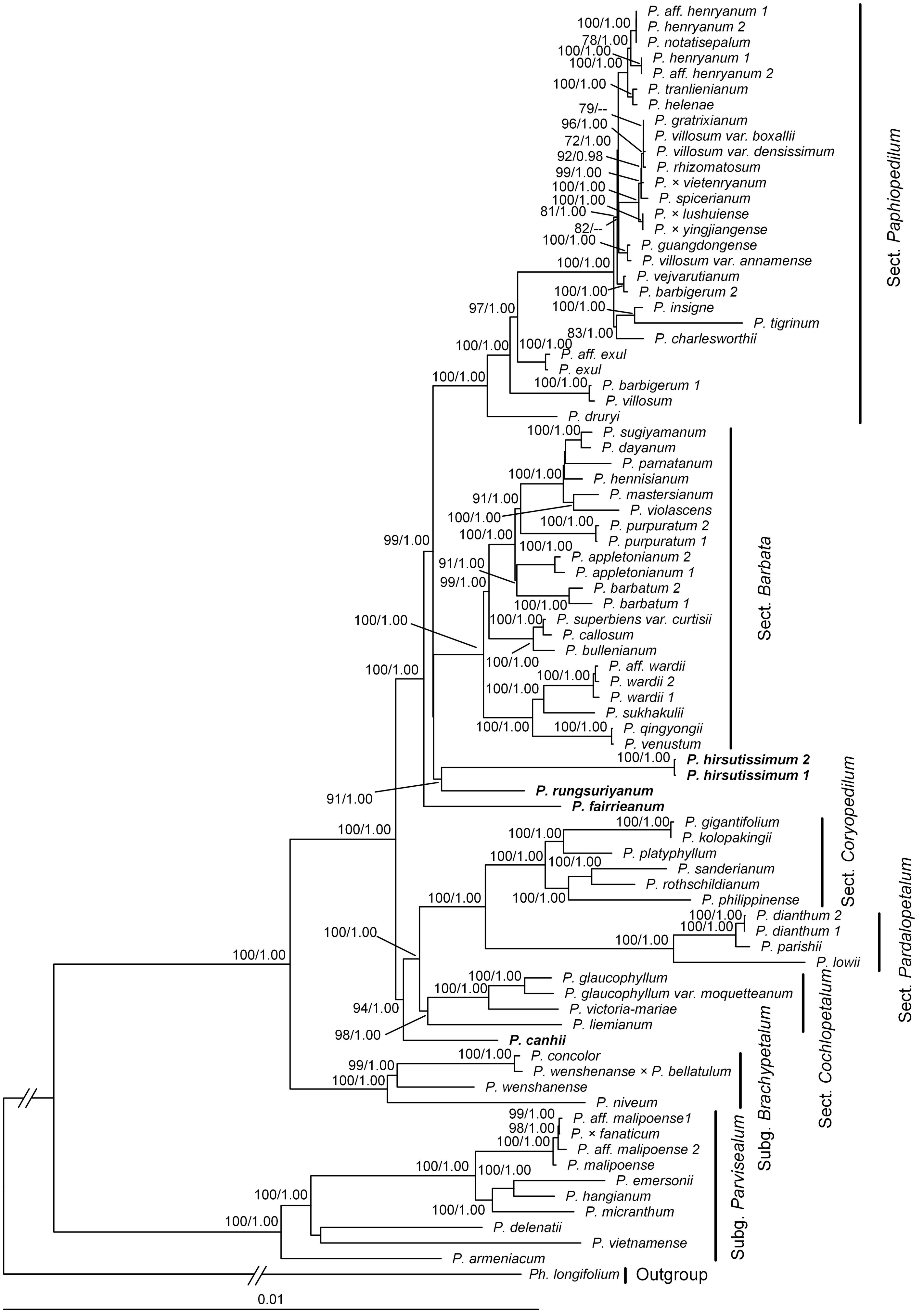

Fig. S3. Phylogenetic tree (ML) of *Paphiopedilum* based on whole plastomes. The number above the branches are the bootstrap values ≥70 and Bayesian posterior probabilities ⩾0.90. The branched in bold are the four unstable species.

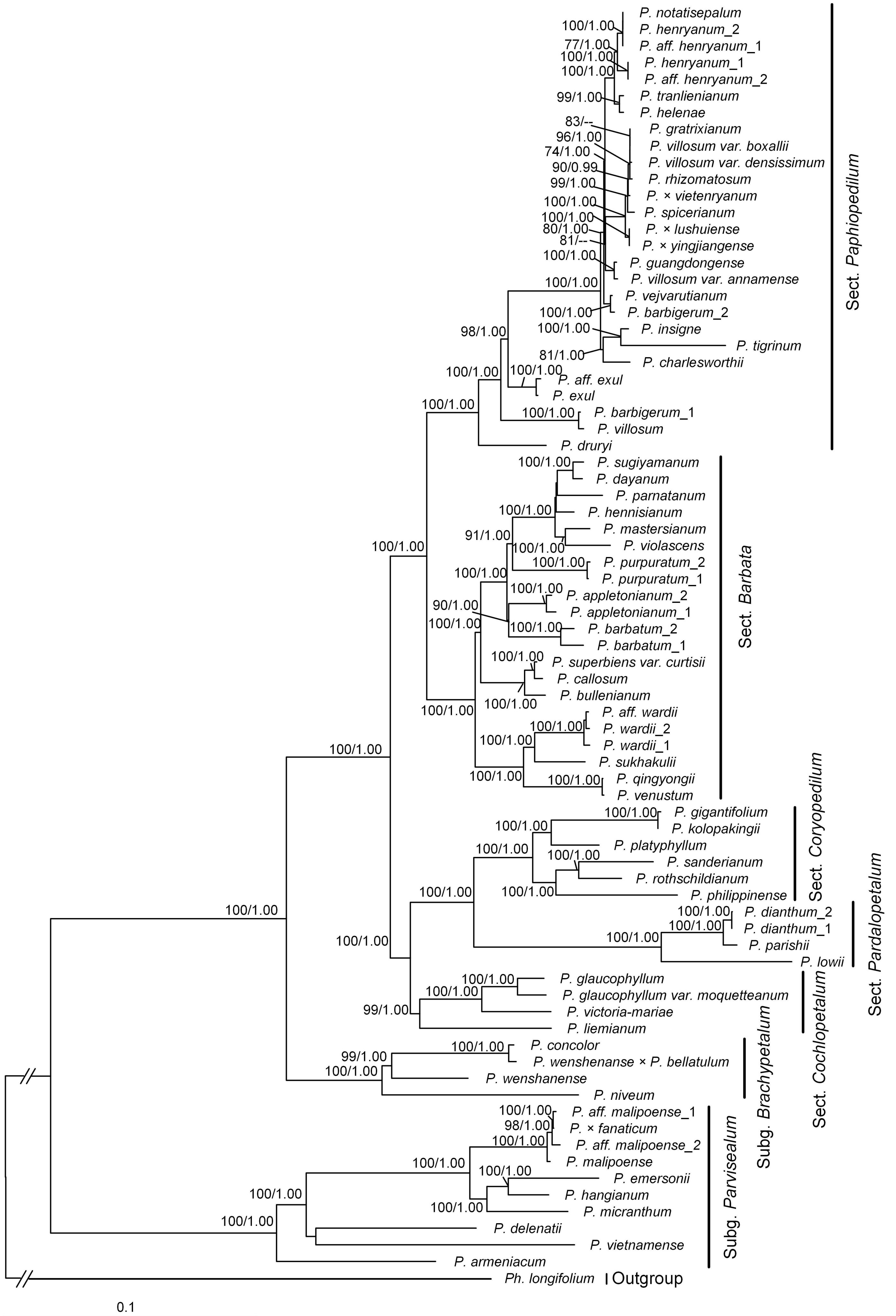

Fig. S3. Phylogenetic tree (ML) of *Paphiopedilum* based on whole plastomes with four unstable species excluded. The number above the branches are the bootstrap values ⩾70 and Bayesian posterior probabilities ⩾0.90.
