# Supplementary material for: The chloroplast genome evolution of Venus slipper (Paphiopedilum): IR expansion, SSC contraction, and highly rearranged SSC regions
Source: BMC Plant Biol. 2021 May 31;21:248. doi: 10.1186/s12870-021-03053-y (PMC8165784; doi:10.1186/s12870-021-03053-y)
Supplement: Supplementary file 2 — Additional file 2: Table S1. Details of accessions included in this study. Table S2. List of genes identified in the chloroplast genomes of Paphiopedilum. Table S3. Gene losses and pseudogenes in Paphiopedilum. Table S4. Non-synonymous substitution rate (dN), synonymous substitution rate (dS), and dN/dS for each gene. Table S5. List of intergenic spacer regions used in this study. [file 12870_2021_3053_MOESM2_ESM.pdf]

Table S1. Details of accessions included in this study.

subgenus	Secttion	Species	Sources/ Vouchers	Size (bp)	LSC (bp)	SSC (bp)	IR (bp)	Number of genes	Protein coding genes	tRNA	rRNA	Pseudogenes		GC content (%)	Gene density	Mean coverage	GenBank No.	Assembling methods
Parvisepalum	Parvisepalum	P. armeniacum	—	162682	91942	3668	33536	130	79	38	8	5	ndhJ , ndhK , ndhC , ycf15 *2	35.40	0.80	—	KT388109*	—
Parvisepalum	Parvisepalum	P. delenatii	NOCC	161012	89892	2550	34285	131	79	39	8	5	ndhJ , ndhK , ndhC , ycf15 *2	35.60	0.81	7768	MN587766	CLC Genomics Workbench
Parvisepalum	Parvisepalum	P. emersonii	NOCC	164092	89346	660	37043	134	81	38	8	7	ndhJ , ndhK , ndhC , ycf15 *2, ndhD *2	35.60	0.82	6391	MN587769	CLC Genomics Workbench/NOVOPlasty
Parvisepalum	Parvisepalum	P. × fanaticum	NOCC	156710	87178	5912	31810	126	77	38	8	3	ycf15 *2, ndhD	36.00	0.80	7779	MN587825	NOVOPlasty
Parvisepalum	Parvisepalum	P. hangianum	NOCC	160767	89867	4600	33150	130	78	38	8	6	ndhJ , ndhK , ndhC , ycf15 *2, ndhD	35.80	0.81	7698	MN587777	CLC Genomics Workbench
Parvisepalum	Parvisepalum	P. malipoense	—	158707	89171	5916	31810	129	77	38	8	6	ndhJ , ndhK , ndhC , ycf15 *2, ndhD	36.00	0.81	7691	MN016934*	NOVOPlasty
Parvisepalum	Parvisepalum	P. aff. malipoense 1	NOCC, 5973	156712	87179	5913	31810	127	76	38	8	5	ndhJ , cemA , ycf15 *2, ndhD	36.00	0.81	7693	MN587824	CLC Genomics Workbench/NOVOPlasty
Parvisepalum	Parvisepalum	P. aff. malipoense 2	NOCC	158876	89347	5909	31810	129	77	38	8	6	ndhJ , ndhK , ndhC , ycf15 *2, ndhD	36.00	0.81	6639	MN587823	NOVOPlasty
Parvisepalum	Parvisepalum	P. micranthum	NOCC	163413	89415	1742	36128	133	80	38	8	5	ndhJ , cemA , ycf15 *2, ndhD	35.70	0.82	7806	MN587791	NOVOPlasty
Parvisepalum	Parvisepalum	P. vietnamense	NOCC	158238	88974	5778	31743	129	77	38	8	6	ndhJ , ndhK , ndhC , ycf15 *2, ndhD	36.00	0.82	7463	MN587809	CLC Genomics Workbench
Brachypetalum	Concoloria	P. concolor	NOCC	161972	90901	1887	34592	131	77	38	8	8	ndhJ , ndhK , ndhC , cemA , ycf15 *2, ndhD *2	35.60	0.81	5832	MN587764	CLC Genomics Workbench
Brachypetalum	Concoloria	P. niveum	—	159108	89958	5194	31978	129	77	38	8	6	ndhJ , ndhK , ndhC , ycf15 *2, ndhD	35.70	0.81	—	KJ524105*	—
Brachypetalum	Concoloria	P. wenshanense	NOCC	163331	90592	1869	35435	132	77	38	8	8	ndhJ , ndhK , ndhC , cemA , ycf15 *2, ndhD *2	35.50	0.81	7628	MN587816	CLC Genomics Workbench/NOVOPlasty
Brachypetalum	Concoloria	P. wenshenanse × P. bellatulum	NOCC	161692	90597	1887	34604	131	77	38	8	8	ndhJ , ndhK , ndhC , cemA , ycf15 *2, ndhD *2	35.70	0.81	6048	MN587752	CLC Genomics Workbench
Paphiopedilum	Pardalopetalum	P. dianthum 1	NOCC	154710	86872	2416	32711	129	78	39	8	4	ndhJ , ycf15 *2, ndhD	35.90	0.83	5787	MN587767	CLC Genomics Workbench
Paphiopedilum	Pardalopetalum	P. dianthum 2	—	154699	86861	2416	32711	129	78	39	8	4	ndhJ , ycf15 *2, ndhD	35.90	0.83	—	MF983795*	—
Paphiopedilum	Pardalopetalum	P. lowii	NOCC, 4624	159048	89141	1833	34037	129	78	38	8	5	ndhJ , ycf15 *2, ndhD *2	35.50	0.81	3784	MN587788	CLC Genomics Workbench/NOVOPlasty
Paphiopedilum	Pardalopetalum	P. parishii	NOCC	154692	86866	2446	32690	129	78	39	8	4	ndhJ , ycf15 *2, ndhD	35.90	0.83	2686	MN587822	CLC Genomics Workbench/NOVOPlasty
Paphiopedilum	Cochlopetalum	P. glaucophyllum	NOCC, 4627	161664	89382	2050	35116	129	79	38	8	4	ycf15 *2, ndhD *2	35.60	0.80	6256	MN587773	CLC Genomics Workbench
Paphiopedilum	Cochlopetalum	P. glaucophyllum var. moquetteanum	NOCC, 6180	161412	89014	2106	35146	129	79	38	8	4	ycf15 *2 ndhD *2	35.60	0.80	3979	MN587774	CLC Genomics Workbench/NOVOPlasty
Paphiopedilum	Cochlopetalum	P. liemianum	NOCC, 6316	~161679	~89515	2342	34911	129	79	38	8	4	ycf15 *2, ndhD *2	35.40	0.80	3622	MN587786	CLC Genomics Workbench
Paphiopedilum	Cochlopetalum	P. victoria-mariae	NOCC, 6322	161277	89340	2225	34856	129	79	38	8	4	ycf15 *2, ndhD *2	35.50	0.80	6310	MN587807	CLC Genomics Workbench/NOVOPlasty
Paphiopedilum	Coryopedilum	P. gigantifolium	NOCC, 2801	159289	87797	2098	34697	129	78	38	8	5	ndhJ , ycf15 *2, ndhD *2	35.60	0.81	2554	MN587772	CLC Genomics Workbench
Paphiopedilum	Coryopedilum	P. kolopakingii	NOCC, 6321	159296	87804	2098	34697	129	78	38	8	5	ndhJ , ycf15 *2, ndhD *2	35.60	0.81	2098	MN587785	CLC Genomics Workbench/NOVOPlasty
Paphiopedilum	Coryopedilum	P. philippinense	NOCC	159237	87430	2147	34830	129	78	38	8	5	ndhJ , ycf15 *2 ndhD *2	35.60	0.81	3385	MN587794	CLC Genomics Workbench
Paphiopedilum	Coryopedilum	P. platyphyllum	NOCC, 4622	160156	88280	2086	34895	129	76	38	8	7	ndhJ , ycf15 *2, ndhD *2, ndhB *2	35.50	0.81	7230	MN587795	CLC Genomics Workbench
Paphiopedilum	Coryopedilum	P. rothschildianum	NOCC, 6071	159045	87471	2154	34710	129	78	38	8	5	ndhJ , ycf15 *2, ndhD *2	35.70	0.81	3723	MN587798	CLC Genomics Workbench/NOVOPlasty
Paphiopedilum	Coryopedilum	P. sanderianum	NOCC, 6324	157240	87475	3867	32949	128	78	38	8	4	ndhJ , ycf15 *2, ndhD	35.70	0.81	7702	MN587799	CLC Genomics Workbench/NOVOPlasty
Paphiopedilum	Paphiopedilum	P. barbigerum 1	NOCC, 9321	160735	89018	677	35520	130	81	38	8	3	cemA , ndhD *2	35.30	0.81	4024	MN587758	CLC Genomics Workbench
Paphiopedilum	Paphiopedilum	P. barbigerum 2	NOCC	~157887	~87074	1281	34766	129	80	38	8	3	cemA , ndhD *2	35.90	0.82	2929	MN587821	CLC Genomics Workbench
Paphiopedilum	Paphiopedilum	P. charlesworthii	NOCC	156806	86539	1827	34220	129	79	39	8	3	cemA , ndhD *2	36.00	0.82	3173	MN587763	CLC Genomics Workbench
Paphiopedilum	Paphiopedilum	P. druryi	NOCC, 6177	158715	88281	1808	34313	128	79	38	8	3	cemA , ndhD *2	35.70	0.81	3721	MN587768	CLC Genomics Workbench
Paphiopedilum	Paphiopedilum	P. exul	NOCC, 9820	159678	88483	1841	34677	129	78	39	8	4	ndhJ , cemA , ndhD *2	35.50	0.81	4637	MN587770	CLC Genomics Workbench
Paphiopedilum	Paphiopedilum	P . aff. exul	NOCC	159671	88548	1831	34646	129	79	39	8	3	cemA , ndhD *2	35.50	0.81	5979	MN587820	CLC Genomics Workbench
Paphiopedilum	Paphiopedilum	P. gratrixianum	NOCC	~157372	~87332	1828	34106	128	79	38	8	3	cemA , ndhD *2	35.80	0.81	5179	MN587775	CLC Genomics Workbench
Paphiopedilum	Paphiopedilum	P. guangdongense	NOCC	156918	86934	1854	34065	128	79	38	8	3	cemA , ndhD *2	35.90	0.82	4882	MN587776	CLC Genomics Workbench/NOVOPlasty
Paphiopedilum	Paphiopedilum	P. helenae	NOCC, 7322	156532	86489	1829	34107	128	78	38	8	4	ndhJ , cemA , ndhD *2	35.90	0.82	4206	MN587778	CLC Genomics Workbench
Paphiopedilum	Paphiopedilum	P. henryanum 1	NOCC	156939	86623	1876	34220	128	78	38	8	4	ndhJ , cemA , ndhD *2	35.90	0.82	3330	MN587780	CLC Genomics Workbench
Paphiopedilum	Paphiopedilum	P. henryanum 2	NOCC	156978	86710	1828	34220	128	78	38	8	4	ndhJ , cemA , ndhD *2	35.90	0.82	1951	MN587781	CLC Genomics Workbench
Paphiopedilum	Paphiopedilum	P. aff. henryanum 1	NOCC, 9287	156978	86710	1828	34220	128	78	38	8	4	ndhJ , cemA , ndhD *2	35.90	0.82	5517	MN587818	CLC Genomics Workbench
Paphiopedilum	Paphiopedilum	P. aff. henryanum 2	NOCC, 9349	~157082	~86766	1876	34220	128	78	38	8	4	ndhJ , cemA , ndhD *2	35.90	0.81	3423	MN587819	CLC Genomics Workbench
Paphiopedilum	Paphiopedilum	P. insigne	NOCC	157398	87021	1901	34238	128	79	38	8	3	cemA , ndhD *2	35.80	0.81	5357	MN587784	CLC Genomics Workbench
Paphiopedilum	Paphiopedilum	P. × lushuiense	NOCC	157029	87013	1828	34094	128	79	38	8	3	cemA , ndhD *2	35.90	0.82	5107	MN587789	CLC Genomics Workbench
Paphiopedilum	Paphiopedilum	P. notatisepalum	NOCC, 9329	156978	86710	1828	34220	128	78	38	8	4	ndhJ , cemA , ndhD *2	35.90	0.82	2299	MN587792	CLC Genomics Workbench/NOVOPlasty
Paphiopedilum	Paphiopedilum	P. rhizomatosum	NOCC	157266	87778	1828	33830	128	79	38	8	3	cemA , ndhD *2	35.80	0.81	3852	MN587797	CLC Genomics Workbench

Paphiopedilum	*Paphiopedilum*	*P. spicerianum*	NOCC	157433	87378	1843	34106	128	79	38	8	3	*cemA* , *ndhD* *2	35.80	0.81	5202	MN587800	CLC Genomics Workbench
Paphiopedilum	*Paphiopedilum*	*P. tigrinum*	NOCC	152130	85113	1873	32572	128	76	39	8	5	*cemA* , *ndhD* *2, *ndhB* *2	35.80	0.84	3919	MN587804	CLC Genomics Workbench
Paphiopedilum	*Paphiopedilum*	*P. tranlienianum*	NOCC, 6359	156504	86468	1834	34101	129	79	38	8	4	*ndhJ* , *cemA* , *ndhD* *2	35.90	0.82	4065	MN587805	CLC Genomics Workbench
Paphiopedilum	*Paphiopedilum*	*P. vejvarutianum*	NOCC	157868	87064	1268	34768	130	80	39	8	3	*cemA* , *ndhD* *2	35.90	0.82	3426	MN587787	CLC Genomics Workbench/NOVOPlasty
Paphiopedilum	*Paphiopedilum*	*P.* × *vietenryanum*	NOCC, 4649	157080	87029	1835	34108	128	79	38	8	3	*cemA* , *ndhD* *2	35.80	0.81	5515	MN587808	CLC Genomics Workbench
Paphiopedilum	*Paphiopedilum*	*P. villosum*	NOCC, 6363	160744	89015	677	35526	130	81	38	8	3	*cemA* , *ndhD* *2	35.30	0.81	3766	MN587810	CLC Genomics Workbench
Paphiopedilum	*Paphiopedilum*	*P. villosum* var. *annamense*	NOCC, 6360	157139	86939	1854	34173	128	79	38	8	3	*cemA* , *ndhD* *2	35.90	0.81	7760	MN587813	CLC Genomics Workbench
Paphiopedilum	*Paphiopedilum*	*P. villosum* var. *boxallii*	NOCC, 5883	157292	87252	1828	34106	128	79	38	8	3	*cemA* , *ndhD* *2	35.80	0.81	7582	MN587812	CLC Genomics Workbench
Paphiopedilum	*Paphiopedilum*	*P. villosum* var. *densissimum*	NOCC	157305	87265	1828	34106	128	79	38	8	3	*cemA* , *ndhD* *2	35.80	0.81	5223	MN587811	CLC Genomics Workbench/NOVOPlasty
Paphiopedilum	*Paphiopedilum*	*P.* × *yingjiangense*	NOCC	157211	87195	1828	34094	128	79	38	8	3	*cemA* , *ndhD* *2	35.80	0.81	6014	MN587817	CLC Genomics Workbench
Paphiopedilum	*Barbata*	*P. appletonianum* 1	NOCC	159670	87982	664	35512	129	80	38	8	3	*ndhJ* , *ndhD* *2	35.30	0.81	7305	MN587755	CLC Genomics Workbench
Paphiopedilum	*Barbata*	*P. appletonianum* 2	NOCC	159029	87891	924	35107	128	79	38	8	3	*ndhJ* , *ndhD* *2	35.40	0.80	6927	MN587756	CLC Genomics Workbench
Paphiopedilum	*Barbata*	*P. barbatum* 1	NOCC	157811	87049	2122	34320	127	78	38	8	3	*ndhJ* , *ndhD* *2	35.60	0.80	6414	MN587757	CLC Genomics Workbench
Paphiopedilum	*Barbata*	*P. barbatum* 2	NOCC	155252	87514	2150	32794	127	76	38	8	5	*ndhJ* , *ndhD* *2, *ndhB* *2	35.40	0.82	6347	MN587750	CLC Genomics Workbench
Paphiopedilum	*Barbata*	*P. bullenianum*	NOCC	160492	89380	2242	34435	128	78	38	8	4	*ndhJ* , *cemA* , *ndhD* *2	35.20	0.80	7017	MN587759	CLC Genomics Workbench/NOVOPlasty
Paphiopedilum	*Barbata*	*P. callosum*	NOCC	160801	89566	2201	34517	128	78	38	8	4	*ndhJ* , *cemA* , *ndhD* *2	35.10	0.80	3914	MN587761	CLC Genomics Workbench
Paphiopedilum	*Barbata*	*P. dayanum*	NOCC	~162020	~91133	2125	34381	127	76	38	8	5	*ndhJ* , *ndhD* *2, *ndhB* *2	34.70	0.78	5104	MN587765	CLC Genomics Workbench
Paphiopedilum	*Barbata*	*P. hennisianum*	NOCC	156863	86347	1886	34315	127	78	38	8	3	*ndhJ* , *ndhD* *2	35.70	0.81	7411	MN587779	CLC Genomics Workbench
Paphiopedilum	*Barbata*	*P. mastersianum*	NOCC, 4614	156580	86916	1986	33839	127	78	38	8	3	*ndhJ* , *ndhD* *2	35.60	0.81	5584	MN587790	CLC Genomics Workbench
Paphiopedilum	*Barbata*	*P. parnatanum*	NOCC, 6315	157339	86722	1921	34348	127	78	38	8	3	*ndhJ* , *ndhD* *2	35.60	0.81	4589	MN587793	CLC Genomics Workbench
Paphiopedilum	*Barbata*	*P. purpuratum* 1	NOCC	~158880	~88450	1462	34484	127	78	38	8	3	*ndhJ* , *ndhD* *2	35.30	0.80	3860	MN587754	CLC Genomics Workbench
Paphiopedilum	*Barbata*	*P. purpuratum* 2	NOCC	~158719	~88259	1462	34499	127	78	38	8	3	*ndhJ* , *ndhD* *2	35.40	0.80	1485	MN587751	CLC Genomics Workbench
Paphiopedilum	*Barbata*	*P. qingyongii*	NOCC	~158523	~88000	949	34787	128	79	38	8	3	*ndhJ* , *ndhD* *2	35.40	0.81	3277	MN587796	CLC Genomics Workbench
Paphiopedilum	*Barbata*	*P. wardii* 1	NOCC	~159004	~88230	1896	34439	127	78	38	8	3	*ndhJ* , *ndhD* *2	35.20	0.80	4183	MN587815	CLC Genomics Workbench
Paphiopedilum	*Barbata*	*P. wardii* 2	NOCC	158839	88103	1908	34414	127	78	38	8	3	*ndhJ* , *ndhD* *2	35.30	0.80	5842	MN587760	CLC Genomics Workbench
Paphiopedilum	*Barbata*	*P.* aff. *wardii*	NOCC, 8713	158769	87999	1906	34432	127	78	38	8	3	*ndhJ* , *ndhD* *2	35.30	0.80	5890	MN587749	CLC Genomics Workbench
Paphiopedilum	*Barbata*	*P. sugiyamanum*	NOCC, 4612	~162112	~91440	2254	34209	127	76	38	8	5	*ndhJ* , *ndhD* *2, *ndhB* *2	34.70	0.78	3105	MN587801	CLC Genomics Workbench
Paphiopedilum	*Barbata*	*P. sukhakulii*	NOCC, 4613	158912	87729	2167	34508	127	78	38	8	3	*ndhJ* , *ndhD* *2	35.30	0.80	3374	MN587802	CLC Genomics Workbench/NOVOPlasty
Paphiopedilum	*Barbata*	*P. superbiens* var. *curtisii*	NOCC, 6797	160153	89512	2171	34235	128	78	38	8	4	*ndhJ* , *cemA* , *ndhD* *2	35.30	0.80	7624	MN587803	CLC Genomics Workbench
Paphiopedilum	*Barbata*	*P. venustum*	NOCC	~158335	~87812	949	34787	128	79	38	8	3	*ndhJ* , *ndhD* *2	35.40	0.81	4690	MN587806	CLC Genomics Workbench
Paphiopedilum	*Barbata*	*P. violascens*	NOCC, 6323	159161	88846	2141	34087	127	78	38	8	3	*ndhJ* , *ndhD* *2	35.20	0.80	5849	MN587814	CLC Genomics Workbench/NOVOPlasty
		P. canhii	NOCC	160788	89842	2236	34355	129	78	38	8	5	*ndhJ* , *ycf15* *2, *ndhD* *2	35.20	0.80	7612	MN587762	CLC Genomics Workbench/NOVOPlasty
		P. fairrieanum	NOCC, 6179	161065	89697	1990	34689	128	78	38	8	4	*ndhJ* , *cemA* , *ndhD* *2	35.20	0.79	3434	MN587771	CLC Genomics Workbench/NOVOPlasty
		P. hirsutissimum 1	NOCC	154889	85057	524	34654	128	79	38	8	3	*cemA* , *ndhD**2	36.30	0.83	2350	MN587782	CLC Genomics Workbench/NOVOPlasty
		P. hirsutissimum 2	NOCC	154878	85062	524	34646	128	79	38	8	3	*cemA* , *ndhD* *2	36.30	0.83	6081	MN587783	CLC Genomics Workbench
		P . rungsuriyanum	NOCC	159198	88158	1848	34596	128	79	38	8	3	*cemA* , *ndhD* *2	35.60	0.80	7768	MN587753	CLC Genomics Workbench/NOVOPlasty

* Sequences downloaded from GenBank.

NOCC: The National Orchid Conservation Center (The Orchid Conservation & Research Center of Shenzhen)

MN016934 was reassembled and reannotated

~sequences with gaps in the LSC regions

— data not available

Table S2. List of genes identified in the chloroplast genomes of *Paphiopedilum* .

Category of genes	Group of genes	Name of genes
**Self-replication**	Ribosomal RNA genes	*rrn4.5* , *rrn5* , *rrn16* , *rrn23*
	Transfer RNA genes	*trnC* -*GCA* , *trnD* -*GUC* , *trnE* -*UUC* , *trnF* -*GAA* , *trnG* -*UCC* [a], *trnG* -*GCC* , *trnK* -*UUU* [a], *trnL* -*UAA* [a], *trnL* -*UAG* , *trnM* -*CAU* , *trnP* -*UGG* , *trnQ* -*UUG* , *trnR* -*UCU* , *trnS* -*GCU* , *trnS* -*GGA* , *trnS* -*UGA* , *trnT* -*UGU* , *trnT* -*GGU* , *trnV* -*UAC* [a], *trnY* -*GUA* , *trnW* -*CCA* , *trnfM* -*CAU* , *trnA* -*UGC* [a] , *trnH* -*GUG* , *trnI* -*CAU* , *trnI* -*GAU* [a], *trnL* -*CAA* , *trnN* -*GUU* , *trnR* -*ACG* , *trnV* -
	Ribosomal protein(small subunit)	*rps2* , *rps3* , *rps4* , *rps7* , *rps8* , *rps11* , *rps12* [b], *rps14* , *rps15* , *rps16* [a], *rps18* , *rps19*
	Ribosomal protein(large subunit)	*rpl2* [a] , *rpl14* , *rpl16* [a], *rpl20* , *rpl22* , *rpl23* , *rpl32* , *rpl33* , *rpl36*
	RNA polymerase	*rpoA* , *rpoB* , *rpoC1* [a], *rpoC2*
	Translational initiation factor	*infA*
**Genes for photosynthesis**	Subunits of photosystem I	*psaA* , *psaB* , *psaC* , *psaI* , *psaJ* , *ycf3* [b], *ycf4*
	Subunits of photosystem II	*psbA* , *psbB* , *psbC* , *psbD* , *psbE* , *psbF* , *psbH* , *psbI* , *psbJ* , *psbK* , *psbL* , *psbM* , *psbN* , *psbT* , *psbZ*
	Subunits of cytochrome	*petA* , *petB* [a], *petD* [a], *petG* , *petL* , *petN*
	Subunits of ATP synthase	*atpA* , *atpB* , *atpE* , *atpF* [a], *atpH* , *atpI*
	Large subunit of Rubisco	*rbcL*
	Subunits of NADH dehydrogenase	*ndhB* [a] , *ndhC* , *ndhD* , *ndhJ* , *ndhK*
**Other genes**	Maturase	*matK*
	Envelope membrane protein	*cemA*
	Subunit of acetyl-CoA	*accD*
	Synthesis gene	*ccsA*
	ATP-dependent protease	*clpP* [b]
	Component of TIC complex	*ycf1*
**Genes of unknown function**	Conserved open reading frames	*ycf2* , *ycf15*

[a] With one intron

[b] With two introns

Table S3. Gene losses and pseudogenes in *Paphiopedilum* .

Subgenus	Secttion	Species	*ndhA*	*ndhB*	*ndhC*	*ndhD*	*ndhE*	*ndhF*	*ndhG*	*ndhH*	*ndhI*	*ndhJ*	*ndhK*	*cemA*	*ycf15*
Parvisepalum	*Parvisepalum*	*P. armeniacum*	−	+	Ψ	−	−	−	−	−	−	Ψ	Ψ	+	Ψ
Parvisepalum	*Parvisepalum*	*P. delenatii*	−	+	Ψ	−	−	−	−	−	−	Ψ	Ψ	+	Ψ
Parvisepalum	*Parvisepalum*	*P. emersonii*	−	+	Ψ	Ψ	−	−	−	−	−	Ψ	Ψ	+	Ψ
Parvisepalum	*Parvisepalum*	*P.* × *fanaticum*	−	+	−	Ψ	−	−	−	−	−	−	−	+	Ψ
Parvisepalum	*Parvisepalum*	*P. hangianum*	−	+	Ψ	Ψ	−	−	−	−	−	Ψ	Ψ	+	Ψ
Parvisepalum	*Parvisepalum*	*P. malipoense*	−	+	Ψ	Ψ	−	−	−	−	−	Ψ	Ψ	+	Ψ
Parvisepalum	*Parvisepalum*	*P.* aff. *malipoense* 1	−	+	−	Ψ	−	−	−	−	−	Ψ	−	Ψ	Ψ
Parvisepalum	*Parvisepalum*	*P.* aff. *malipoense* 2	−	+	Ψ	Ψ	−	−	−	−	−	Ψ	Ψ	+	Ψ
Parvisepalum	*Parvisepalum*	*P. micranthum*	−	+	Ψ	Ψ	−	−	−	−	−	Ψ	Ψ	+	Ψ
Parvisepalum	*Parvisepalum*	*P. vietnamense*	−	+	Ψ	Ψ	−	−	−	−	−	Ψ	Ψ	+	Ψ
Brachypetalum	*Concoloria*	*P. concolor*	−	+	Ψ	Ψ	−	−	−	−	−	Ψ	Ψ	Ψ	Ψ
Brachypetalum	*Concoloria*	*P. niveum*	−	+	Ψ	Ψ	−	−	−	−	−	Ψ	Ψ	+	Ψ
Brachypetalum	*Concoloria*	*P. wenshanense*	−	+	Ψ	Ψ	−	−	−	−	−	Ψ	Ψ	Ψ	Ψ
Brachypetalum	*Concoloria*	*P. wenshenanse* × *P. bellatulum*	−	+	Ψ	Ψ	−	−	−	−	−	Ψ	Ψ	Ψ	Ψ
Paphiopedilum	*Pardalopetalum*	*P. dianthum* 1	−	+	−	Ψ	−	−	−	−	−	Ψ	−	−	Ψ
Paphiopedilum	*Pardalopetalum*	*P. dianthum* 2	−	+	−	Ψ	−	−	−	−	−	Ψ	−	−	Ψ
Paphiopedilum	*Pardalopetalum*	*P. lowii*	−	+	−	Ψ	−	−	−	−	−	Ψ	−	−	Ψ
Paphiopedilum	*Pardalopetalum*	*P. parishii*	−	+	−	Ψ	−	−	−	−	−	Ψ	−	−	Ψ
Paphiopedilum	*Cochlopetalum*	*P. glaucophyllum*	−	+	−	Ψ	−	−	−	−	−	+	−	−	Ψ
Paphiopedilum	*Cochlopetalum*	*P. glaucophyllum* var. *moquetteanun*	−	+	−	Ψ	−	−	−	−	−	+	−	−	Ψ
Paphiopedilum	*Cochlopetalum*	*P. liemianum*	−	+	−	Ψ	−	−	−	−	−	+	−	−	Ψ
Paphiopedilum	*Cochlopetalum*	*P. victoria-mariae*	−	+	−	Ψ	−	−	−	−	−	+	−	−	Ψ
Paphiopedilum	*Coryopedilum*	*P. gigantifolium*	−	+	−	Ψ	−	−	−	−	−	Ψ	−	−	Ψ
Paphiopedilum	*Coryopedilum*	*P. kolopakingii*	−	+	−	Ψ	−	−	−	−	−	Ψ	−	−	Ψ
Paphiopedilum	*Coryopedilum*	*P. philippinense*	−	+	−	Ψ	−	−	−	−	−	Ψ	−	−	Ψ
Paphiopedilum	*Coryopedilum*	*P. platyphyllum*	−	**Ψ**	−	**Ψ**	−	−	−	−	−	**Ψ**	−	−	Ψ
Paphiopedilum	*Coryopedilum*	*P. rothschildianum*	−	+	−	Ψ	−	−	−	−	−	Ψ	−	−	Ψ
Paphiopedilum	*Coryopedilum*	*P. sanderianum*	−	+	−	Ψ	−	−	−	−	−	Ψ	−	−	Ψ
Paphiopedilum	*Paphiopedilum*	*P. barbigerum* 1	−	+	−	Ψ	−	−	−	−	−	+	−	Ψ	−
Paphiopedilum	*Paphiopedilum*	*P. barbigerum* 2	−	+	−	Ψ	−	−	−	−	−	+	−	Ψ	−
Paphiopedilum	*Paphiopedilum*	*P. charlesworthii*	−	+	−	Ψ	−	−	−	−	−	+	−	Ψ	−
Paphiopedilum	*Paphiopedilum*	*P. druryi*	−	+	−	Ψ	−	−	−	−	−	+	−	Ψ	−
Paphiopedilum	*Paphiopedilum*	*P. exul*	−	+	−	Ψ	−	−	−	−	−	Ψ	−	Ψ	−
Paphiopedilum	*Paphiopedilum*	*P* . aff. *exul*	−	+	−	Ψ	−	−	−	−	−	+	−	Ψ	−
Paphiopedilum	*Paphiopedilum*	*P. gratrixianum*	−	+	−	Ψ	−	−	−	−	−	+	−	Ψ	−
Paphiopedilum	*Paphiopedilum*	*P. guangdongense*	−	+	−	Ψ	−	−	−	−	−	+	−	Ψ	−
Paphiopedilum	*Paphiopedilum*	*P. helenae*	−	+	−	Ψ	−	−	−	−	−	Ψ	−	Ψ	−
Paphiopedilum	*Paphiopedilum*	*P. henryanum* 1	−	+	−	Ψ	−	−	−	−	−	Ψ	−	Ψ	−
Paphiopedilum	*Paphiopedilum*	*P. henryanum* 2	−	+	−	Ψ	−	−	−	−	−	Ψ	−	Ψ	−
Paphiopedilum	*Paphiopedilum*	*P.* aff. *henryanum* 1	−	+	−	Ψ	−	−	−	−	−	Ψ	−	Ψ	−
Paphiopedilum	*Paphiopedilum*	*P.* aff. *henryanum* 2	−	+	−	Ψ	−	−	−	−	−	Ψ	−	Ψ	−
Paphiopedilum	*Paphiopedilum*	*P. insigne*	−	+	−	Ψ	−	−	−	−	−	+	−	Ψ	−
Paphiopedilum	*Paphiopedilum*	*P.* × *lushuiense*	−	+	−	Ψ	−	−	−	−	−	+	−	Ψ	−
Paphiopedilum	*Paphiopedilum*	***P. notatisepalum***	−	+	−	Ψ	−	−	−	−	−	Ψ	−	Ψ	−
Paphiopedilum	*Paphiopedilum*	*P. vejvarutianum*	−	+	−	Ψ	−	−	−	−	−	+	−	Ψ	−
Paphiopedilum	*Paphiopedilum*	*P. rhizomatosum* ?	−	+	−	Ψ	−	−	−	−	−	+	−	Ψ	−
Paphiopedilum	*Paphiopedilum*	*P.* × *vietenryanum*	−	+	−	Ψ	−	−	−	−	−	+	−	Ψ	−
Paphiopedilum	*Paphiopedilum*	*P. villosum*	−	+	−	Ψ	−	−	−	−	−	+	−	Ψ	−
Paphiopedilum	*Paphiopedilum*	*P. villosum* var. *annamense*	−	+	−	Ψ	−	−	−	−	−	+	−	Ψ	−
Paphiopedilum	*Paphiopedilum*	*P. villosum* var. *boxallii*	−	+	−	Ψ	−	−	−	−	−	+	−	Ψ	−

Paphiopedilum	*Paphiopedilum*	*P. villosum* var. *densissimum*	−	+	−	Ψ	−	−	−	−	−	+	−	Ψ	−
Paphiopedilum	*Paphiopedilum*	*P. spicerianum*	−	+	−	Ψ	−	−	−	−	−	+	−	Ψ	−
Paphiopedilum	*Paphiopedilum*	*P. tigrinum*	−	Ψ	−	Ψ	−	−	−	−	−	−	−	Ψ	−
Paphiopedilum	*Paphiopedilum*	*P. tranlienianum*	−	+	−	Ψ	−	−	−	−	−	Ψ	−	Ψ	−
Paphiopedilum	*Paphiopedilum*	*P.* × *yingjiangense*	−	+	−	Ψ	−	−	−	−	−	+	−	Ψ	−
Paphiopedilum	*Barbata*	*P. appletonianum* 1	−	+	−	Ψ	−	−	−	−	−	Ψ	−	−	−
Paphiopedilum	*Barbata*	*P. appletonianum* 2	−	+	−	Ψ	−	−	−	−	−	Ψ	−	−	−
Paphiopedilum	*Barbata*	*P. barbatum* 1	−	+	−	Ψ	−	−	−	−	−	Ψ	−	−	−
Paphiopedilum	*Barbata*	*P. barbatum* 2	−	Ψ	−	Ψ	−	−	−	−	−	Ψ	−	−	−
Paphiopedilum	*Barbata*	*P. bullenianum*	−	+	−	Ψ	−	−	−	−	−	Ψ	−	Ψ	−
Paphiopedilum	*Barbata*	*P. callosum*	−	+	−	Ψ	−	−	−	−	−	Ψ	−	Ψ	−
Paphiopedilum	*Barbata*	*P. dayanum*	−	Ψ	−	Ψ	−	−	−	−	−	Ψ	−	−	−
Paphiopedilum	*Barbata*	*P. hennisianum*	−	+	−	Ψ	−	−	−	−	−	Ψ	−	−	−
Paphiopedilum	*Barbata*	*P. mastersianum*	−	+	−	Ψ	−	−	−	−	−	Ψ	−	−	−
Paphiopedilum	*Barbata*	*P. parnatanum*	−	+	−	Ψ	−	−	−	−	−	Ψ	−	−	−
Paphiopedilum	*Barbata*	*P. purpuratum* 1	−	+	−	Ψ	−	−	−	−	−	Ψ	−	−	−
Paphiopedilum	*Barbata*	*P. purpuratum* 2	−	+	−	Ψ	−	−	−	−	−	Ψ	−	−	−
Paphiopedilum	*Barbata*	*P. qingyongii*	−	+	−	Ψ	−	−	−	−	−	Ψ	−	−	−
Paphiopedilum	*Barbata*	*P. sugiyamanum*	−	Ψ	−	Ψ	−	−	−	−	−	Ψ	−	−	−
Paphiopedilum	*Barbata*	*P. sukhakulii*	−	+	−	Ψ	−	−	−	−	−	Ψ	−	−	−
Paphiopedilum	*Barbata*	*P. superbiens* var. *curtisii*	−	+	−	Ψ	−	−	−	−	−	Ψ	−	Ψ	−
Paphiopedilum	*Barbata*	*P. wardii* 1	−	+	−	Ψ	−	−	−	−	−	Ψ	−	−	−
Paphiopedilum	*Barbata*	*P. wardii* 2	−	+	−	Ψ	−	−	−	−	−	Ψ	−	−	−
Paphiopedilum	*Barbata*	*P.* aff. *wardii*	−	+	−	Ψ	−	−	−	−	−	Ψ	−	−	−
Paphiopedilum	*Barbata*	*P. venustum*	−	+	−	Ψ	−	−	−	−	−	Ψ	−	−	−
Paphiopedilum	*Barbata*	*P. violascens*	−	+	−	Ψ	−	−	−	−	−	Ψ	−	−	−
		P. canhii	−	+	−	Ψ	−	−	−	−	−	Ψ	−	−	Ψ
		P. fairrieanum	−	+	−	Ψ	−	−	−	−	−	Ψ	−	Ψ	−
		P. hirsutissimum 1	−	+	−	Ψ	−	−	−	−	−	−	−	Ψ	−
		P. hirsutissimum 2	−	+	−	Ψ	−	−	−	−	−	−	−	Ψ	−
		P. *rungsuriyanum*	−	+	−	Ψ	−	−	−	−	−	+	−	Ψ	−

'+' indicates presence of the gene, 'Ψ' indicates pseudo-copy of the gene, and '−' indicates complete absence of the gene.

Table S4. Non-synonymous substitution rate (dN), synonymous substitution rate (dS), and dN/dS for each gene.

Gene	Complex	dN	dS	dN/dS
atpH	ATP_synthase	0.0153	0.0485	0.31622
atpB	ATP_synthase	0.0186	0.0801	0.23253
atpI	ATP_synthase	0.0325	0.102	0.3187
atpA	ATP_synthase	0.0351	0.1002	0.34967
atpE	ATP_synthase	0.0095	0.1264	0.07542
atpF	ATP_synthase	0.0881	0.142	0.62044
petG	Cytochrome	0	0.0532	0.0001
petL	Cytochrome	0.0142	0.0515	0.27596
petN	Cytochrome	0.0151	0.1067	0.14177
petB	Cytochrome	0.0107	0.1113	0.09595
petD	Cytochrome	0.017	0.1488	0.11444
petA	Cytochrome	0.0323	0.1567	0.20642
psaJ	Photosystem_1	0	0.0695	0.0001
psaC	Photosystem_1	0	0.0825	0.0001
psaA	Photosystem_1	0.0261	0.0859	0.30337
psaB	Photosystem_1	0.0263	0.1102	0.23895
psaI	Photosystem_1	0.0488	0.1261	0.3869
psbJ	Photosystem_2	0.0109	0.0424	0.25619
psbZ	**Photosystem_2**	**0.037**	**0.0235**	**1.57679**
psbC	Photosystem_2	0.0311	0.0572	0.54468
psbH	**Photosystem_2**	**0.0482**	**0.0467**	**1.0318**
psbB	Photosystem_2	0.019	0.0942	0.20218
psbD	Photosystem_2	0.0198	0.0938	0.2114
psbE	Photosystem_2	0.0053	0.1147	0.04587
psbN	Photosystem_2	0.0327	0.0919	0.35598
psbF	Photosystem_2	0	0.144	0.0001
psbA	Photosystem_2	0.0088	0.1367	0.06443
psbL	Photosystem_2	0.0103	0.1436	0.07148
psbK	Photosystem_2	0.0531	0.1116	0.47564
psbT	Photosystem_2	0	0.1759	0.0001
psbI	Photosystem_2	0.1225	0.1544	0.79342
psbM	Photosystem_2	0.3585	0.7519	0.47682
rpl23	Ribosomal_large	0.005	0.015	0.3314
rpl2	Ribosomal_large	0.0208	0.0271	0.76819
rpl16	Ribosomal_large	0.0396	0.0523	0.7579
rpl14	Ribosomal_large	0.0576	0.149	0.3869
rpl33	Ribosomal_large	0.0751	0.1761	0.42657
rpl36	Ribosomal_large	0.0364	0.2183	0.16685
rpl22	Ribosomal_large	0.0785	0.195	0.40229
rpl20	Ribosomal_large	0.1947	0.219	0.88913
rpl32	Ribosomal_large	0.1222	0.3193	0.3827
rpoB	RNA_polymerase	0.0393	0.1274	0.3082
rpoC1	RNA_polymerase	0.034	0.155	0.21945

rpoC2	RNA_polymerase	0.0603	0.1294	0.46627
rpoA	RNA_polymerase	0.0557	0.1577	0.3534
rps19	Ribosomal_small	0.014	0.0184	0.76416
rps12	Ribosomal_small	0.0038	0.0323	0.11756
rps7	Ribosomal_small	0.0172	0.0382	0.45147
rps14	Ribosomal_small	0.0261	0.0573	0.45541
rps2	Ribosomal_small	0.039	0.0731	0.53278
rps16	Ribosomal_small	0.0425	0.0983	0.43252
rps15	Ribosomal_small	0.0388	0.1183	0.32758
rps4	Ribosomal_small	0.0632	0.1042	0.60669
rps11	Ribosomal_small	0.0465	0.128	0.36347
rps8	Ribosomal_small	0.0788	0.1032	0.76383
rps18	Ribosomal_small	0.0725	0.1366	0.53032
rps3	Ribosomal_small	0.0587	0.2422	0.24246
ycf3	ORF	0.0157	0.0366	0.43014
ycf2	ORF	0.0266	0.029	0.91804
ycf4	ORF	0.0482	0.1158	0.41629
ycf1	ORF	0.0821	0.114	0.72051
rbcL	other	0.0073	0.0862	0.0842
infA	other	0.0325	0.1713	0.1896
accD	other	0.0946	0.1334	0.70906
clpP	**other**	**0.1675**	**0.1012**	**1.65609**
matK	Maturase	0.1183	0.1701	0.6955
ccsA	other	0.0932	0.2543	0.36664

Table S5. List of intergenic spacer regions used in this study.

Intergenic spacer regions	Aligned length(bp)
psbA-trnK(UUU)	327
trnK(UUU)-rps16	1621
rps16-trnQ(UUG)	1412
trnQ(UUG)-psbK	493
psbK-psbI	819
psbI-trnS(GCU)	172
trnS(GCU)-trnG(UCC)	2530
trnG(UCC)-trnR(UCU)	159
trnR(UCU)-atpA	716
atpA-atpF	88
atpF-atpH	406
atpH-atpI	947
atpI-rps2	381
rps2-rpoC2	299
rpoC2-rpoC1	198
rpoC1-rpoB	26
rpoB-trnC(GCA)	1730
trnC(GCA)-petN	1485
petN-psbM	3154
psbM-trnD(GUC)	923
trnD(GUC)-trnY(GUA)	403
trnY(GUA)-trnE(UUC)	76
trnE(UUC)-trnT(GGU)	3267
trnT(GGU)-psbD	1208
psbC-trnS(UGA)	174
trnS(UGA)-psbZ	248
psbZ-trnG(GCC)	320
trnG(GCC)-trnfM(CAU)	199
trnfM(CAU)-rps14	184
rps14-psaB	209
psaB-psaA	25
psaA-ycf3	1109
ycf3-trnS(GGA)	661
trnS(GGA)-rps4	314
rps4-trnT(UGU)	636
trnT(UGU)-trnL(UAA)	1457
trnL(UAA)-trnF(GAA)	431
trnV(UAC)-trnM(CAU)	290
trnM(CAU)-atpE	236
atpB-rbcL	2186
rbcL-accD	974
accD-psaI	1135
psaI-ycf4	408
petA-psbJ	1307
psbJ-psbL	153
psbL-psbF	22

psbF-psbE	10
psbE-petL	954
petL-petG	208
petG-trnW(CCA)	134
trnW(CCA)-trnP(UGG)	179
trnP(UGG)-psaJ	2403
psaJ-rpl33	780
rpl33-rps18	715
rps18-rpl20	310
rpl20-rps12	880
rps12-clpP	197
clpP-psbB	1972
psbB-psbT	1857
psbT-psbN	82
psbN-psbH	110
psbH-petB	254
petB-petD	205
petD-rpoA	233
rpoA-rps11	100
rps11-rpl36	211
rpl36-infA	125
infA-rps8	191
rps8-rpl14	1338
rpl14-rpl16	189
rpl16-rps3	198
rps3-rpl22	207
rpl22-rps19	358
rps19-trnH(GUG)	146
trnH(GUG)-rpl2	51
rpl2-rpl23	18
rpl23-trnI(CAU)	223
trnI(CAU)-ycf2	68
trnV(GAC)-rrn16	229
rrn16-trnI(GAU)	308
trnI(GAU)-trnA(UGC)	64
trnA(UGC)-rrn23	145
rrn23-rrn4.5	98
rrn4.5-rrn5	228
rrn5-trnR(ACG)	260
trnR(ACG)-trnN(GUU)	517
ycf1-trnN(GUU)	517
